# Clinical Review and Hospital Reports

**Published:** 1835-01-01

**Authors:** 


### Clinical Review.

### St. George's Hospital.

### Abstract of a Lecture on Injuries of the Nerves, delivered by Mr. Cæsar Hawkins.

We are indebted to the kindness of Mr. Hawkins for the following highly interesting remarks on an interesting subject. The lecture from which they are extracted is one of those delivered by Mr. Hawkins in the school of St. George's Hospital.

The next class of injuries which we have to consider are those which are inflicted on *nerves*, which form a very curious and interesting subject, in consequence of modern investigations into the connexions and functions of these parts. Every case perhaps will present to you some new and peculiar feature, and hence probably it happens, that no practical division of them has been made with which I am acquainted; and yet I think it will be found that there are some general effects resulting from injuries of nerves, which usually allow of their being conveniently placed under two heads, according as the nerve has been *partially injured* only, or *totally divided*. I do not know that such a distinction has anywhere been drawn, but I think that, as a general rule, it will hold good, and suffice to guide you towards the proper remedial agent.

1st, then, as a general rule, partial injury or slighter bruises, or moderate pressure, will be followed by excited circulation in the nerve, inflammation, acute sensibility, and increased heat, spasm of muscles, and other evidence of *augmented nervous energy*.

2dly. Complete division of a nerve, or great pressure, will usually be attended with loss of function in the injured nerve—diminished temperature—paralysis of the muscles—loss of sensibility, and impaired vitality in the parts which are thus cut off from their natural connexion with the sensorium; unless, thirdly, inflammation be induced subsequently to the injury, in which the symptoms of the two sets of cases will be mixed.

1. The excitement of a nerve from partial injury may be confined almost entirely to the situation of the injury, or to the parts supplied by the nerve, or the effects may be more extensive, reaching to the origin of the nerve in the spinal marrow or the brain, or in a higher degree, still, the whole body may be deranged by the injury, and the powers of the mind weakened and impaired.

A young woman came to me with violent pain in a small cicatrix, which appeared to involve the dorsal branch of the ulnar nerve, the pain sometimes reaching the shoulder; the cicatrix itself was exquisitely tender, and pressure on any part of the ulnar nerve, even in the axillary plexus, caused considerable inconvenience. By a blister to the spine and purgatives the pain was removed except from the cicatrix, and this also was subsequently cured by belladonna liniment, and by galbanum pills with colocynth.

A young woman was in the hospital under the care of Sir B. Brodie some years ago, who had been bitten in the back of the forearm by a dog two months previously. This gave her violent pain for three days, and continued more or less during the whole time. For a fortnight before her admission the head had swelled, with greater pain in the cicatrix and in the head;—she could bend her fingers but not extend them, and could not use her thumb at all, which was the most painful, and nearest to the cicatrix, which seemed to involve a part of the external cutaneous nerve;—and within the last day or two the pain had begun to extend towards the axilla. Sir B. Brodie cut out the painful cicatrix, and all the symptoms were at once removed.

In an interesting paper by Mr. Earle, in the Med. Chir. Trans. on the influence of the nerves on animal temperature, he relates an instance in which a prick of the external cutaneous nerve, half-way down the forearm, was followed by repeated attacks of local inflammation, with the large bullæ of Pemphigus, and the heat of the part was actually three degrees higher than under the tongue. In these cases the irritation of a cutaneous nerve was the cause of nearly local symptoms only, but if the system be disordered, the effects of an injury even of a superficial branch may be nearly universal.

A woman was in the hospital, who had been bled in the forearm. The operation was attended with much pain from an injury, no doubt, of a cutaneous nerve. The pain shortly extended below and above the cicatrix;—soon the fingers and hand became contracted and immoveable—spasms extended upwards to the neck and head—soon afterwards the whole of the same side of the body became affected with convulsive spasms—and on her admission even the opposite side of the body was partially affected in the same way. This woman's digestive organs were much deranged, and by attention to their functions the nervous symptoms were almost entirely removed. Several cases resembling the last have been related by Mr. Abernethy, which were produced like that by bleeding.

2. Partial injuries of the deeper and muscular nerves are succeeded by the same evidence of excitement as when the smaller and superficial branches only are inflamed. A woman was under my care in the hospital, who had partially divided the ulnar nerve close to the wrist by breaking a glass window. The wound quickly healed, and I desired her to return to me if she felt anything unusual in the hand. A month afterwards she came back, complaining of violent pain on the ulnar edge of the hand, especially on the back of the metacarpal bone of the little finger, and the whole course of the ulnar nerve and the axillary plexus above the clavicle were exquisitely tender and painful. She could not move the fingers, and the temperature of the ulnar side of the hand was higher than natural. She attended for a long time as an outpatient before I could afford her any relief.

A very interesting case has been related by Dr. Denmark of an officer, who was wounded at the siege of Badajoz in the arm. This gentleman suffered the most excruciating pain and burning, which were aggravated by the least motion, and the elbow and wrist were contracted and bent; he had violent starting of the limb, and suffered from frightful dreams. The little finger however was free from pain, and a small tumor was felt in the median nerve at the bottom of the wound. The arm was amputated, and a small piece of the ball was found embedded in the median nerve.

I have thus related to you several cases of partial injury, no two of which, you will perceive, are exactly alike, although they all agree in presenting evidence of either general or local *nervous excitement.*

If the symptoms are nearly confined to the injured part, local means alone will sometimes enable you to relieve the patient—cold lotions, such as linen dipped in æther or spirit of wine—or opiates, such as infusion of tobacco or solution of opium, a drachm in eight ounces of water—or a belladonna plaster—or a liniment composed of one-third of extract of belladonna, with two-thirds of spermaceti or other cerate. Sometimes a few leeches are desirable when the temperature is high, and after this has been reduced, without entire subsidence of the pain, a small blister, or tartar emetic ointment.

May the affected portion of nerve be removed by operation as in one of the cases I have related? Certainly it may, when there is a cicatrix or tumor shewing the exact seat of the pain, provided 1st, that the nerve is a *superficial* one, for the operation must not be performed where a deep or large and muscular nerve is affected; as the remedy is worse than the disease, for reasons which will presently be evident to you. And provided, 2dly, that the pain and other symptoms are *local* only, without *general affection* of the system; for if the general health be deranged the operation is highly improper. Either the principal cause is in the general health, and by attention to this cause, whatever it may be, the local symptoms may be subdued, and the removal of the portion of nerve rendered unnecessary; or, if such an operation be performed before the general health is corrected, the same symptoms will in a short time return in connexion with the end of the nerve nearest to the brain, or by branches of communication being formed between the two ends of the nerve, or between the lower end of the divided nerve and some other neighbouring branch—or else, and perhaps more frequently, if there be much excitement, the patient's sufferings may be immediately aggravated in degree, or by the addition of new symptoms.

If, then, in any case of nervous excitement from partial injuries, the general health is disturbed, you must endeavour to ascertain whether the disease has originated in the derangement of some particular function—the digestive—or uterine—or if the patient be only of that peculiarly nervous temperament which is usually called hysterical, and you must correct these different conditions just as you would in a case of tic douloureux, without injury of a nerve. Most frequently the digestive functions are improperly or inadequately performed; let your patient take therefore 5 or 10 grains of comp. aloetic pill every night, or 10 grains of aloetic pill with myrrh twice daily—or an ounce of comp. decoction of aloes with five grains of carbonate of ammonia, and some camphor mixture or light bitter—or let him take 3 grains of blue-pill and 8 or 10 of extract of rhubarb every night, with light bitter infusion of calumba or cascarilla, with infusion of senna twice a day—or some ammonia or carbonate of potassa with bitter medicines, or with bark.

After a course of purgatives, if they are required, steel has great power—an ounce of steel mixture, or half a drachm of carbonate or tartrate of iron twice or thrice daily, or 10 grs. of comp. steel pill with 5 of myrrh, and aloe pill three times a day, in conjunction with a shower-bath or cold bath.

Sometimes arsenic relieves the state of system on which the nervous symptoms partly depend, from three to eight drops of liquor arsenicalis thrice daily, with some camphor mixture or aromatic, or with decoction and extract of sarsaparilla, or with some alkali added, magnesia or solution of potassa, where the common form of arsenic disagrees. Sometimes one or two grains of sulphate of zinc with extract of hemlock thrice a day, or some oxyde of bismuth will be of service.

Now and then the pain requires narcotics; acetate of morphia or opium when it is severe, and occasionally, but not very often, the symptoms will yield to the extracts of stramonium or belladonna internally, from one to three grains of stramonium, or from half a grain to a grain of belladonna three times in the day. I have much less faith, however, in the internal administration of opiates than I have in their external use. In fact, all the local applications I have already mentioned are to be conjoined with internal means.

But, further, in some cases you may do much good by occasionally cupping or leeching on the part of the spine from which the affected nerve arises, and which is often tender and painful. Even a blister or belladonna plaster is sometimes of more service on the loins or neck than on the affected nerve itself, though I am not inclined to look for the cause of nervous pains in this part of the body, so often as some modern authors would lead you to do.

3. I remarked just now that, if a portion of nerve was cut out, the same symptoms frequently returned at the upper end of the nerve; that is to say, besides the more immediate effects of complete division of a nerve, you may have irritation established with vascular excitement in the part nearest to the brain. This however is chiefly in the smaller and superficial nerves, and the curious though well-known phenomenon is then observed of the reference of the pain by the patient to the parts originally supplied by the nerve, so that pain may be apparently excited in the toes or fingers years after a limb has been removed by amputation. This circumstance will be generally in cases where, after amputation, the end of the bone has exfoliated, or the stump has, from any other cause, been kept in a state of inflammation, or the nerve is involved in the cicatrix, and the case exactly resembles one of excited action, from partial injury, and requires just the same attention for the removal of the symptoms.

But, further, the consequence of long-continued irritation in any injured part of a nerve, when entire, or in the extremity of a divided nerve, produces a bulbous enlargement, with a separation of the nervous filaments from one another, by deposit of organized lymph into the neurilema, the tumor which is thus produced being exquisitely tender to the touch, and the pain produced by it being so severe, as seriously to impair the patient's health. Here is a preparation in which you may see all the nerves of the arm in this condition, after amputation above the elbow, the end of the humerus having a small piece of dead bone attached to it. Here, again, is the same appearance at the upper end of the median nerve, which had been divided by a wound across the fore-arm. Where, then, such tumors form after amputation, a second amputation is sometimes necessary, for the same reasons, and with the same precautions, as when a partially-divided nerve is involved in a cicatrix. Mr. Langstaff has lately published a paper, in which he attributes the occurrence of these bulbous tumors of the nerves in a stump to the circular method of performing the operation, and thinks it can be avoided by the flap operation. I do not think, however, that there is any reason for supposing that it is the result of any thing more than inflammation, and, therefore, that in a healthy person, and a well-formed stump, it is not likely to take place from one operation more frequently than from the other; it may, indeed, be a reason for shortening a large nerve, whenever there seems any danger of its adhering to the line of union. It is, however, but a rare circumstance, either in amputation, or in any other injury of nerves.

4. If a ligature is tied tightly round a nerve, the same effect is produced upon the parts which are supplied by the nerve as if it had been wholly divided; but the influence upon the nerve itself is the same as when inflammation takes place, either in a partially-divided nerve, or in one which has been cut across, but has been subjected to inflammatory action afterwards. The effect, therefore, will be to cause much local or general irritation, and, at last, a bulbous enlargement forms, as the result of the inflammation upon the upper part of the nerve. It is somewhat remarkable that, in such a case, the tumor does not form on both sides of the ligature, though the nerve might be expected, from analogy with other structures, to inflame equally, above and below, the irritating cause; but, as far as I know, it is produced in that portion only which is connected with the brain or spinal marrow. A very interesting case is related by Dr. Hennen, illustrating the effects of ligatures on nerves, in the person of a General Officer, whose arm was amputated, and several nerves of the axillary plexus tied with the arteries, the ligatures being, of course, a long time before they could be detached. The pulling one of these ligatures would cause pain to be referred to the fingers of the amputated member—of another, in the thumb, or in the elbow, or in the wrist. The pulling of one ligature would not always cause pain in the same part, but the supposed situation of the pain would vary, according to the manner or the direction in which it was moved, so that, if it was dragged downwards, the pain might be referred to the fingers, but if inwards, to the elbow;—and if it was dragged roughly, the whole of the lost limb would seem to be in much pain, and pain would extend upwards to the head, with fainting and general suffering.

5. But let us suppose that a large nerve has been wholly divided, without there having been any local irritation or general derangement of the system of the patient to occasion inflammation in the nerve, like that which almost always attends partial injuries of this structure. The effects of such a division will be immediate and remote. As soon as the injury has been inflicted, there is necessarily an instant deprivation of sense and voluntary power in the parts supplied by the nerve, so that, if nothing more remarkable took place, the affected part would become wholly useless, and would shrink and waste for want of exertion.

It would appear, from the experiment of Haighton and Cruikshank, and other physiologists, that when a nerve is simply divided, the cut ends will unite, and the functions of the part would be restored after some time; and such is doubtless the case with the smaller nerves at least, so that, when a nerve has been divided in tic douloureux, or other nervous pains, the part, though numbed for a time, soon regains sensation, and the disease consequently returns again. If you examine a nerve, which has thus united after union, you will perceive, indeed, that the structure of the uniting medium is not like nerve, but that the nervous energy is transmitted through a tough cellular, or half ligamentous structure, not very unlike the substance which unites the divided ends of muscular fibres, though not so dense. It is very curious, however, that not only is this new substance capable of transmitting nervous influence from the upper to the lower end of a divided small nerve, though not itself possessing the appearance of nervous matter, but even when a nerve has had a considerable portion actually cut out, a restoration of power may take place, partly by the intervening cellular substance, but in part, also, from the actual formation of new nervous fibrils, shot out from the divided ends and uniting them together, or forming anastomoses with some neighbouring nerve. In the person who had this bulb, formed upon the upper part of a divided median nerve, there was an interval of full three inches between the two ends, for the lower end had been turned down in a loop towards the wrist, where it had been fixed, and yet the muscles of the thumb and fingers seemed to have regained their power, because some small anastomosing branches had been formed, which came across from the superficial branch of the muscular spiral nerve. Mr. Swan verified this fact in several cases, in which he had divided the cutaneous nerves of the leg for very painful varices of the veins; and general experience in the operations which have been performed for tic douloureux of the fifth nerve, in the face, proves the restoration of sensation, and the inefficiency of all such operations, except as temporary palliatives. But, although the case I have just mentioned would appear to shew, that a nerve, of the size of the median nerve, may have the functions of the parts supplied by it restored in these two ways, yet unquestionably you must not expect it in the larger nerves, except in some very rare instances; there is, in general, no return either of sense or motion, or, occasionally, the sensation may be partially restored, while the locomotive power is not in the least recovered. It would seem, too, as if, in any nerve, the power of regeneration is less likely in man, than in other animals on whom experiments have been instituted.

If the nervous influence of a part has been destroyed by *pressure only*, as by dislocation of the clavicle or humerus, even then a very long time will sometimes elapse before the patient can again use the arm, the restoration being, however, sometimes hastened by friction, by blisters, or tartar-emetic ointment, or electricity. But if it is lost by entire division of a large nerve, the case is hopeless; and, if the extremity have only one nerve, as the ischiatic or popliteal, the patient drags a useless limb, which is an actual incumbrance that amputation alone can remove. You will find some interesting observations by Mr. Guthrie upon this point, in one of his works—that on Gun-shot Wounds I think.

But not only are sense and motion lost by the entire interruption of the course of nervous influence—there result also from such an injury some more remote effects, which are highly interesting. Such a case as this is very common, and shews you that the division of even a small nerve becomes the source of a good deal of distress and inconvenience to the patient. A man came into the hospital under my care, in consequence of a wound of the wrist, which he had produced by some broken glass, and which, besides other unimportant injury, had cut across that part of the median nerve which supplies the fingers, leaving those branches entire which supply the thumb. There was, of course, instant deprivation of sense in the fore and middle fingers, and in one side of the ring finger, and even the power of motion was in some measure impaired. The wound quickly healed, however, and he left the hospital; but about a month afterwards, the weather happening to become cold, he returned, because two fingers had inflamed, and the cuticle and nails had separated, and portions even of the cutis had sloughed. This mischief was quickly repaired, however, but he remained constantly liable to the same inflammation.

In fact, then, the effects of entire division of nerves is a loss of the power of regulating *the heat* of the parts which have lost their nervous energy, and a diminution of their vital powers. They are incapable of resisting heat and cold, and they inflame and die from injuries which would not be felt if the nerves were not divided or interrupted. The patient I have just alluded to produced sloughing, by washing his hands in some by no means very cold water, and any one whose hand or fingers are in the same condition cannot use either hot or cold water—he cannot handle a piece of metal or marble, or any other good conductor of heat, the temperature of which is at all higher or lower than his hand; he cannot warm his hand at the fire, or do any action of this kind without destruction to the parts, which cannot regulate their supply of animal heat in the usual manner. At the same time, the vitality of the parts is materially lowered, independent of the effects of changes of temperature, so that bruises or pressure which can be borne with impunity in a healthy part, are followed by ulceration, and sloughing, and abscess. All these effects of loss of nervous influence are seen, on a large scale, in cases of injuries or diseases of the spinal marrow, where the patient is so frequently carried off by sloughing or abscesses of the nates or legs, or by inflammation of the bladder, and where the injury to the nervous influence produces, also, some other very curious effects, which, from their greater importance, and from their being more complicated, are more interesting still than the simple effects of the loss of power in a single nerve. Of these I shall have to speak in a future lecture.

These singular phenomena are not to be attributed to loss of sensation only, rendering the patient incapable of guarding against pressure or changes of temperature, of the existence of which he is not aware; but the vitality is *actually lowered*, and the effects are, therefore, invariable and constant, and you must always caution your patients strictly against exposure to any of those circumstances which may act upon the injured parts.

It appears to me, moreover, that the effects I am describing to you depend, for the most part, upon the *sensitive* portion only of the nerve, and not upon the motive part. The veterinary surgeon knows that his horse, the *cutaneous* nerve of whose foot has been cut to relieve it from a painful disease, will always slough and ulcerate, so as more than to balance any advantages derived from the operation. In M. Majendie's experiment of cutting the root of the fifth nerve, the nerve of sensation to the head, the surface of the eye in a few days ulcerates, and the eye is destroyed. And I have had occasion to point out to you the phenomena in question, as a mode of diagnosis between disease of the spinal marrow and of the bones of the spine, *i. e.* if there is sloughing and loss of temperature, the disease is probably in the spinal marrow, since, in ordinary caries of the spine, the disease being in the bodies only of the vertebræ, the irritation is not propagated to more than the anterior part of the spinal marrow, which is nearest to the seat of the caries, and, therefore, although there may be total paraplegia, the functions of the posterior half of the spinal marrow are unimpaired. So, again, in hemiplegia, dependent on disease of the brain, you do not meet with sloughing, and there is very little difference in the power of regulating the temperature of the affected half of the body.

Observe in these curious cases, too, this fact.—The temperature is not only adjusted to surrounding objects, but it is *actually lowered* considerably below the natural standard. I have only seen it eight degrees below what it should be, but it is sometimes much more than this; in the paper by Mr. Earle, in the Med.-Chirurgical Transactions, which I have already alluded to, he mentions a case in which the affected arm was 70° only, while the other was at 92°, a difference, it will be observed, of 22°. And this case shewed the want of regulating power in this way; they were both subjected to electricity, but while the heat of the sound arm was not raised, but remained stationary at 92°, the temperature of the paralysed arm was elevated from 70° to 77°.

In this respect, there is generally a great contrast between partial injuries, in which there is almost always inflammation, and cases of total division, in which inflammation is rare, except at first; in the case of inflamed nerve, therefore, the temperature of parts supplied by the nerve is as many degrees higher than the natural standard, as, in the other case, it is below what it ought to be. The analogy between injuries of the spinal marrow, and of the nerves derived from it, is here, also, kept up, as the temperature of the parts below the injury is generally several degrees above that of the upper part of the body; I am not aware, however, that the temperature of the parts below an inflamed nerve is ever higher than that of the interior of the body; while I have seen it, in a case of injury of the spine, as high as 111° just before death.

It would appear, too, that these effects upon the vitality of a part are as permanent as they are curious; many years, at all events, elapse, and leave the patient still suffering from them.

What I have thus remarked to you is sufficient, I trust, to shew you the propriety of the limit I mentioned, in operations for painful affections of the nerves, viz. that no good surgeon should think of cutting any nerve, except the small and superficial ones, because the remedy is worse than the disease. It has been done, before surgeons were as well aware of the consequences as they are at the present time, and the effects were just what I have enumerated.

Mr. Earle some years ago cut the ulnar nerve, behind the elbow, in a young woman who had a painful affection of the little finger. She experienced the benefit that was expected from the operation, and was completely relieved from her pain; but then, besides the loss of sensation and of motion, which were anticipated, she suffered, in a way that was not expected, from constant attacks of inflammation in the fore-arm. Five years afterwards, these effects still remained, and the temperature of the little finger was then four degrees lower than that of the other, which was the same difference that existed soon after the operation.

I have very little to say to you as to the treatment of this class of cases, for, unfortunately, we have little power of doing more than cautioning the patient against exposure to changes of temperature, by appropriate clothing, and by avoiding every thing which is likely to excite inflammatory action. A little good is done, if there is pain, by opiate applications and Goulard's extract, and a distressing feeling of numbness is relieved by touching the affected part with camphorated spirit, or sulphuric æther; but there is no room for that variety of remedies which are required, and which are beneficial in the other class of partial injuries, with excited circulation and augmented nervous influence. One curious part of these cases, however, is, that although the parts deprived of their due supply of nervous influence will inflame and slough so easily, yet the sloughs separate, and the ulcers granulate and cicatrize, and the abscesses fill up very readily, so that there is no difficulty, with the usual attention, to repair the mischief which has been produced, unless the vast extent of the sores destroys the strength of the patient, as it so often does with injuries of the spine.

---

CHARING-CROSS HOSPITAL.

SURGICAL REPORT.

FRACTURED RIBS—LACERATION OF THE HAND AND ARM.

Eliz. Wall, æt. 71, a robust woman, of temperate habits, was admitted into the hospital under the care of Mr. Pettigrew Feb. 5th, having been knocked down and ran over by an omnibus, the wheels of which passed over her chest and hand. Two ribs were fractured obliquely under the right breast, and the hand and arm were much lacerated. She complained of much pain at each inspiration, and had a troublesome cough, to which she said she had long been subject. Her pulse was 126 and feeble. A girdle bandage was buckled round her chest to fix the ribs, and the lacerated wounds dressed with adhesive plaster. *Fever diet. Pil. hydrarg. submur.* gr. v. h. s. s. *Haust. cath. mane.*

6th. Had slept but little—cough troublesome—expectoration difficult—respiration easier. Pulse 100 and stronger—bowels open. *Mist. feb,* c̄ *Oxym. scill.*

7th. P. 120 and feeble—has had but little sleep—expectoration still difficult—respiration uneasy—complained of the stiffness of the bandage, for which a flannel roller was substituted.

8th. P. 130. Tongue moist—cough troublesome. *Rep. mist. c. add Tinct. digitalis,* ♏. x. 4*tis horis.*

She continued for the next three days much in the same state; the cough continuing troublesome, and the expectoration scanty. The pulse was reduced to 112, and had more strength. *Omit. Tinct. digit.* On the evening of the 11th her pulse was 120 and very irregular—tongue foul—respiration not very difficult—sloughing of the cellular membrane of the hand. *Catapl. commun.*

12th. Considerable inflammation extending up the arm—had passed a restless night—cough frequent—respiration difficult—pain of side at each inspiration—pulse 92 and irregular—tongue foul — congestion apparent from the blueness of the countenance—urine dark and turbid—has had the bowels relieved twice. *Mist. feb.* ℥iss. *Pulv. ipec. c.* gr. iij. 6*tis horis.* In the evening the pulse was ranging between 128 and 136 and feeble. The sloughing of the hand extending.

13th. Had passed a good night, and was much better—urine copious—pulse 124 and stronger—tongue furred—cough less—respiration easier. No action of the bowels. *Haust. cath. stat.* In the evening pulse 134, tongue brownish, breathing improved, head and arm easy.

14th. Pulse 124—tongue furred—hand very painful, and discharging an ill-conditioned pus—a small slough over the external condyle of the humerus—bowels not relieved. *Rep. haust. cath.* which operated freely during the day.

15th. P. 124 and irregular—tongue still brown and dry, but skin moist—hand and arm easier—respiration difficult, and complains of a sense of tightness in the chest. *Pulv. ipec. c. to be discontinued.*

16th. Pulse 124—tongue white—bowels open—expectoration difficult, and respiration uneasy. *Emulsio pect.* c̄ *Tinct. opii c.*

From this time the urgency of the symptoms abated; the skin became moist; the cough less troublesome, although the expectoration was difficult; the pulse reduced in frequency and gained strength. Healthy granulations of the hand and arm ensued, and on the 22d she was able to be got out of bed. She gradually improved, and was discharged the hospital on March 31st.

FRACTURED RIBS.

Hannah Clarke, æt. 85, was admitted April 26th, under the care of Mr. Pettigrew, having been knocked down by a cabriolet, the wheel of which passed over her back and fractured several of the ribs on the left side. Her breathing was difficult, and attended with much pain. Pulse frequent but feeble.

*Hyd. submur.* gr. v. *stat. Haust cath. hor.* 1 *post.* Roller round the chest. In the evening she complained of great pain of the side—pulse 120 and hard —tongue white—respiration difficult. *V.S. ad* ℥viij. The blood uninflamed. Her pulse sunk to 116, and became much weaker.

27th. Had passed a good night—pulse 108 and soft—respiration natural —no cough—urine copious and natural —bowels not acted upon. *Rep. purgat. Pulv. ipecac. c.* gr. x. *h. s. s.*

28th. Bad night—side painful—slight cough—respiration easy—pulse 112, soft and weak—tongue white and dry —bowels still confined. *Haust. cath. 4tis horis. Enema commune stat. Pulv. ipec. c.* gr. x. *h. s. s.*

29th. The injection had freely relieved the bowels—pulse 104, soft and regular—side painful—had passed a bad night. *Mist. sal.* ℥iss. *4tis horis.*

30th. Cough troublesome—pulse 108 and weak—expectoration frothy—tongue white—bowels relieved twice. *Cont. Remed.*

May 1st. Had passed a better night —pulse 100 and soft—cough slight—expectoration scanty. ℞. *Pulv. rhœi,* gr. vj. *Pulv. ipec. c.* gr. x. *M. ft. pulv. h. s. s. Cont Mist.*

2d. Expectoration slightly tinged with blood—had a tolerable night—p. 108, soft and weak. Bowels much relaxed, and the discharges very offensive. *Cont. remed.*

She continued to improve; the cough varying more or less, and the expectoration diminishing. Union of the fractured ribs proceeded much more rapidly than might have been expected from the advanced age of the patient, and she was well enough to be made an out-patient on the 26th.

### Fractured Femur—no Union—Death.

Mary Canty, æt. about 70, admitted June 27th under the care of Mr. Pettigrew. She was much afflicted with rheumatism. Fell down whilst sweeping her room, and fractured the right thigh. The fracture was oblique and about three inches from the condyles. The limb was readily made to correspond in length with the other, the muscles offering no resistance whatever to the extension made. The limb was put up and laid upon the fracture-board, and was quite easy. At night the muscles were found to have contracted violently, and the fractured ends of the bone were displaced. *Pulv. opii c.* gr. x. *h. s. s. Haust cath. cras mane.* She passed the night badly; had severe pain in both limbs, but more in the sound than in the injured one: the latter was much swollen, and the ends of the bone displaced. The inclined plane was removed, and the limb extended with a pillow beneath the ham. The position gave great ease. *Cold lotion to the thigh. Mist. feb.* ℥iss. *4tis horis.*

29th. Bowels relieved—skin soft—pulse 104—tongue furred. *Pil. sapon.* c̄ *Opio,* gr. v. *h. s. s.* She passed a good night, but the muscles contracted violently, and the bones were again displaced. Opiates were combined with the fever mixture, and all kinds of contrivances were resorted to, to keep the limb steady, but without effect. Dessault's splint was used, but the spasmodic contractions were so violent, that the ends of the bones were continually displaced. The limb was again put upon the double-inclined plane, and splints made firm by rollers, tapes, &c. The contractions, however, in this position occasioned so much pain that the plane was obliged to be taken away, and the limb supported only by pillows. In this way she went on for a fortnight, when ulceration of the integuments of the back took plack, and sloughing commenced. She was placed upon the hydrostatic bed, and the usual dressings resorted to. Her sufferings, however, were great, and nothing like union or any attempt at union appeared to have taken place. Her appetite failed, and her powers began to sink: aphthous appearances ensued, and she sunk on the 16th October, having been nearly four months in the hospital. Examination of the femur shewed the fracture to have been oblique, and the bones were overlapping each other to the extent of three inches and a half. The fractured surfaces were moveable upon each other, and the edges as sharp as at the moment of the accident. No bony matter had been thrown out; some condensed cellular membrane was beginning to form a kind of ligamentous connexion along the sides of the fractured bone.

### Compound Fracture of the Tibia and Fibula—Death.

John Atwell, æt. 63, was admitted into the hospital July 1st, under the care of Mr. Pettigrew, with a compound fracture of the right leg. When brought into the hospital, the lower portion of the tibia protruded through the integuments, about three inches from the ankle-joint. There was considerable hæmorrhage, the vena saphena major being wounded. The bone was replaced, and the wound dressed; the bleeding arrested, and the leg put loosely up in Sharpe's splints. The patient was of a full habit of body, of a very irritable temper, had led an irregular life, and was labouring under a fistula in ano, for which he had been operated upon, but the wound had not healed, in consequence of his urine making its escape through the wound, there being a communication between that part and the urethra. *Haust. cath. stat.—Cold lotion to the leg—Fever diet.*

2d. Feverish, and has had rigors several times during the night, but very little pain of the leg. *Mist. feb.* ℥jss. *4tis horis.*

On the 8th, the dressings were removed, and the wound, which was about the size of half-a-crown, looked healthy. It was then dressed daily, the discharge became copious, and the pus was occasionally unhealthy, and mixed with blood; sinuses were beginning to form in different directions. He was put upon a more generous diet, and the pus, by being carefully pressed out at each dressing, soon became healthier, and less in quantity. Healthy granulations formed in the wound, which contracted daily. On the 18th of August, it had quite healed. During this period, his health was tolerable—attention was paid to his bowels, as he occasionally needed an anodyne. A slight œdema appeared about the ankle and foot, but was unattended with pain.

On the 29th of August the leg was examined, and it was clearly ascertained that the bones had united. The splints were taken off. He was, however, very weak, and ordered *Dec. cinchon.* ℥ij. c̄ *Acid. sulph. dilut.* ♏x. *ter die.*

Sept. 1st. The leg became very painful; it was œdematous, and there was an erysipelatous blush on the skin. He had passed a bad night—the face was flushed—skin hot and dry—pulse quick—tongue furred—bowels natural. Fomentations were applied, and the antiphlogistic regimen adopted,

3d. He was better, and the inflammation was less, but the œdema extended to the thigh, and affected equally the other limb—urine scanty. By the use of diuretics, the urine was increased and the œdema was reduced, and he was so much better on the 6th as to be able to sit up. He could bear the weight of his body upon the fractured leg. He continued to improve, although both limbs remained much swollen, until the 13th, when he again became feverish—pulse 120, and very feeble—tongue white—bowels relaxed—urine scanty.

From this time he gradually got worse. He had occasional rigors, diarrhœa—deficient urine—his face flushed—both lower extremities and abdomen œdematous—sleepless nights—no appetite—great thirst, and was unable to retain food upon his stomach. He became weaker and weaker, and, on the 23d October, expired. It had been proposed to him to remove from the hospital, to improve his general health, but he was unwilling to quit it. Examination of the body was not permitted, but the leg was inspected. The bones were found to be only partially united—it is probable new bone had been absorbed during the continuance of the diarrhœa and other symptoms, marking the failure of his powers and general disturbance of the system.

### Steatomatous Tumor—Removal.

Ann Ford, æt. 38, was admitted into the hospital July 15th, under the care of Mr. Pettigrew, for a tumor, situated on the upper part of the back, inclining to the right side, and measuring 23 inches in circumference. She stated that it had been attaining its present size during six years; that it first appeared as a small lump on the back of the neck, and grew very gradually until the last year. She enjoyed good health, and Mr. Pettigrew determined upon its removal. Two incisions, in the form of an ellipsis, were made, and the tumor dissected out. The veins were large and numerous, and a large quantity of blood was lost; but not a single artery required the ligature. Mr. P. attributed this, in great part, to a free use of cold water during the operation. The adhesions of the tumor to the surrounding parts were very strong, and great care was requisite to avoid wounding the fascia covering the trapezius muscle. The tumor was steatomatous. The healing of the wound was very favourable, and the woman was well enough to quit the hospital on the 2d of August.

---

### ST. THOMAS'S HOSPITAL.

### NEURALGIA.

Mary Anne Huntingford, a servant, æt. 28, was admitted into St. Thomas's Hospital, under the care of Dr. Roots, April 3d, 1834. She then stated, that she had been subject to paroxysms of pain in the loins, thighs, and legs, for more than two years, which had gradually increased. She had been in the hospital, under Dr. Elliotson, in January last, by whom she was at first freely depleted, after which the disease assumed an intermittent form, and she then took large doses of quinine, and was slightly relieved by it, but left the hospital before she was well, and soon became worse.

At the time of her second admission, her general health was not impaired. She was subject to paroxysms of pain, generally two every day, in the loins, hips, thighs, and legs, following the course of the sciatic nerve. The pain was of a severe cutting kind, and attended by some twitching of the muscles. There was tenderness on pressure of the lumbar vertebræ, and along the whole course of the sciatic nerve. The paroxysms of pain came on and ceased suddenly, and without any warning; they did not observe any regular intervals, and were of variable duration, often continuing for several hours; and in the intervals she was seldom free from uneasiness. Has no symptoms of hysteria; menstruation regular.

Quinæ sulph. gr. v.

Ferri sub-carb. ʒij.—6tâ quaq. horâ.

Empl. canth. sacro.

April 9th. No change.

Ferri carb. ʒiij. Extr. stramonii, gr. ss. 6tis horis.

On the 12th the paroxysms were of rather shorter duration, but returned as frequently, and were quite as severe as ever. The quinine was increased to gr. vij. and the iron to ʒiv. in each dose. On the 14th she had no pain, and only a little on the evening of the 16th. Quinine increased to gr. x. in each dose. The intermittent character of the pain continued to the 30th, and the paroxysms had gradually become less severe and of shorter duration. The stramonium was omitted on this day, as the sight had become affected by it.

May 3d. The improvement had continued, and the quinine was increased to gr. xv. From the 9th to the 24th she suffered from headach and sickness, but the medicine was continued, and the pain abated. On the 24th the iron was increased to ʒvj. and on the 28th gr. ¼ of muriate of morphia was ordered with every dose.

June 11th. The pain still better, and intermitting, but, in consequence of continued headach and sickness, all the medicines were omitted.

Ung. veratri (℈j. ad ʒj.) ʒj. ter die lumbis.

On the 14th the pain had become more severe, and the quinine, carbonate of iron, and muriate of morphia, were gradually resumed, and increased up to August 23d, at which time she was taking—

Quinæ sulph. ℈j.

Ferri subcarb. ℥j.

Morphiæ muriat. gr. ⅓, 6tis horis.

These medicines were omitted for four days, during which she took some creosote; but the pains returned immediately after the medicine was changed, and continued to increase. The old medicines were resumed, and she quickly improved, as before. She left the hospital on the 22d Sept. of her own accord; the pain had diminished much in severity, returned much less frequently, and not at any regular intervals.

The pain soon increased, but never attained its original severity. She was again admitted into the hospital under Dr. Roots, Nov. 21st. The pain was of the same character, and in the same situation, as before; commencing in the loins, and afterwards affecting the gluteal muscles, and extending down the back part of the thighs to the hams and heels. The paroxysms usually came on about 7, p.m. and continued four or five hours. During the attack the muscles were firm and contracted. There was some tenderness of the gluteal muscles at all times. General health good; pulse 75, feeble.

On the 25th, the following ointment was ordered :—

Aconitæ, gr. ij.

Ung. cetacei, ʒj., ft. ung. et infricet pars sexta part. dolent nocte maneq.

On the 28th, she stated that after each application of the ointment, the parts rubbed became hot and smarted, but this was quickly followed by numbness. After the third application the pain was a little relieved, and after the fifth, the amendment was very remarkable. The paroxysms were much diminished in severity, and did not continue more than one or two hours. The pain, which had formerly been very acute, she described as being much less severe, and called it a " burning twitching." The parts were less tender on pressure, and she could sit up without causing pain in the gluteal muscles, which she could not do three days since.

Infricet. unguent. ter die.

Dec. 2d. Two days since, in the morning, she had a more violent paroxysm of pain than she has had since her admission. After it had continued an hour and a half, the ointment was applied, and in ten minutes she was much relieved. The paroxysms do not now last more than twenty minutes, and she says they have never been so slight before.

6th. The pain has now resumed its intermittent character. On the 3d, 5th, and 7th, she had a short and slight paroxysm in the evening, each being less severe than that preceding it.

Quinæ sulph. gr. v., 6tis horis.

9th. The pain returns every other night, but is gradually decreasing in duration and severity.

There is a man in Luke's Ward, under Dr. Roots, who has a painful affection of the sciatic nerve, for which he used veratrine ointment for some time without any benefit. He has used the aconitine for a week, and a very decided mitigation of the pain followed its application.

Dr. Roots informs me that he has used the same remedy in private practice in three cases with similar success.*

In the foregoing case there was exhibited *bold practice*, in respect to doses. We know Dr. Roots well, and believe him to be a very judicious physician. We should have great confidence in his prescriptions, because we are satisfied that he acts under the guidance of observation and reflection. Eighty grains of sulphate of quinine, added to a quarter of a pound of carbonate of iron, with a strong dose of morphine, in the 24 hours, make, altogether, a "*quantum suff.*" that would astonish a Bonhommie, a Quinn, a Hahnemann—or, indeed, any man who

* Renshaw's Med. and Surg. Journal had not studied in St. Thomas's Hospital. We remember, full well, the sensations which we ourselves experienced, some years ago, when taking twenty grains daily of sulphate of quinine for an intermittent. The tensive feelings about the head—the "tightness" (as Dr. Philip would say) of the pulse—the constriction in the line of the intestinal canal—the *cordiness* (if we might use such an expression) of every nerve—and the painfully vivid operations of the mind—left an impression on the memory which can never be effaced. There can be no doubt that difference of temperament, in individuals, makes immense difference in the effects of medicines; but we have so often remarked effects corresponding with those enumerated, in various individuals, that we confess some degree of timidity in the *heroic* exhibition of medicines. With respect to the carbonate of iron, we suspect that a drachm dose would be just as efficient as a quarter of a pound—perhaps more so. We acknowledge, however, that it is by experience, and not by theoretical calculations, that we are to be guided in these matters; and, therefore, we have put the foregoing case on record, from the practice of a talented physician, in order that it may excite the attention of our readers.

---

### Manchester Royal Infirmary.

The following cases of chorea and of jaundice are taken from the volume of Clinical Reports, lately published by Dr. Carbutt, of Manchester. The press of other matter prevented their insertion in the last Number of this Journal. We alluded to the volume, or rather to its preface, and took the liberty of questioning the propriety of trusting greatly to "principles," in the practical department of medicine. Our attention will now be directed to the "cases," the pabulum alike of doctor and of critic.

A work like this is insusceptible of analysis, and not adapted for a critique. The most natural and most advantageous mode of rendering it useful to our readers will be to bring before them, at convenient opportunities, the leading facts which it contains. Its articles will dovetail, without difficulty, into our clinical department.

### I. Chorea.

*Case* 1. Grace Plant, æt. 24, married, admitted June 22d, 1833.

She first perceived involuntary motions in her limbs about a month ago. She had the sense of a ball, rising up in her throat, which she attributed to wind. Bowels obstinately costive. Pain in the head. Appetite bad. Tongue clean and moist. Has not perceived her catamenia except twice, and then scanty, since her marriage, a period of six years. Has had three children, the youngest is sixteen months old. Since she weaned her child, a fortnight ago, she has felt considerably better. She has lately laboured under much mental anxiety. She says the first symptoms of her affection were brought on by too much thinking, or, as she terms it, too much study. Pulse slow, and rather feeble. Her nose, for the last few days, has slightly bled. Has no pain anywhere except the headache. Has slight lepra on her legs.

To take five grains of the Pilula Aloës cum Myrrhâ, every hour whilst waking.

23rd. Bowels copiously moved. Tongue clean. Appetite good. Cannot sleep. Complains that the rising of wind is very troublesome. No diminution in the involuntary motion of her limbs.

In addition to the pills, to take the warm bath every night.

25th. Perspires much after the warm bath, which she thinks does her good. Leprous affection of her legs much improved. Convulsions less violent. Bowels much purged.

The twitchings now diminished, but on the 28th, she had been much troubled with globus hystericus, and had such pain in the head, that eight leeches were applied to the temples. After this she progressively improved, and on the 15th July, was discharged cured.

*Case* 2. James Lowe, æt. 12, admitted July 1, 1833.

Works in a cotton-factory. Has been ill six months. The convulsions are very strong; he is entirely speechless from the difficulty of articulating; he has much stupor. Bowels are costive. Tongue is foul. He has pain in the head.

To take a pill of five grains of the pilula Aloës cum Myrrhâ every hour whilst waking.

2d. Bowels unmoved.

3d. Has had very copious motions; the stools consist of an immense quantity of feculent matter of all colours. He is rather improved.

The convulsions diminished further, and the motions grew more natural. On the 17th, he was ordered to take the shower-bath every morning, and to persevere with the pills. Some amendment ensued, but, on the 24th, he complained of head-ache.

To omit the bath and the pills—to take Pilula Zinci Sulphatis, gr. j. ter die.

On the 1st of August, the twitchings were not gone.

℞. Liquoris Morphinæ Acetatis, ♏vj.

Misturæ Acidi Sulphurici f ℥iij.

Misce.

To take an ounce after each pill.

After this, he progressively improved; and, on the 19th of August, was discharged cured.

The following judicious remarks of Dr. Carbutt are deserving of insertion.

"In the case before us,* gentlemen, I have principally to remark, that, whereas it is laid down by Sydenham,† that St. Vitus's Dance attacks children of from ten to fourteen years of age, the present patient is twenty-four years of age, and, what is more remarkable, she is a married woman and has had three children. The complication of hysteria with chorea is not to be wondered at, as these two diseases have a considerable resemblance to each other in many particulars. The woman was also affected with lepra vulgaris, but it seems quite uncertain whether or not it was the partial suppression of this leprous affection which gave rise to the two nervous affections.

With regard to the predisposing causes of chorea, they are said to be the feminine gender, the nervous temperament, hereditary liability, infancy, or at any rate, an age before that of puberty. Here, however, we have the curious fact of a married woman with children, she herself being twenty-four years of age, having the disease. The exciting causes of chorea *are said* to be great fright, a fit of anger, violent and repeated crosses, the presence of worms in the alimentary canal, difficulty or suppression of the menses, and suppressed cutaneous eruption.

With regard to the seat and nature of chorea, that is to say, its proximate cause, much has been said and written, with which I do not intend to trouble you; but I will lay before you my own ideas on the subject. The commencement of chorea I believe to be an irritation produced in the intestines either by the presence of accumulated feces, or by the presence of worms. This irritation is conveyed to the brain, and produces uneasiness there. The brain makes an instinctive effort to get rid of this uneasiness; but, having no power over the involuntary muscles of the intestines, it excites disorderly motions in the only muscles over which it has power, the voluntary. These disorderly motions, being once excited, soon become confirmed by habit, which, as you well know, has great power over the voluntary muscles. The disorderly motions of which I speak have been well designated by a French author, *la folie musculaire*, that is to say muscular madness. Now, the consequence of the confirmation of the habit of disorderly motions in the voluntary muscles, is that the motions do not immediately cease upon the removal of the cause

---

* The first case which we have noticed.—Eds.

† 'Schedula monitoria.' sect. 19.

which originally excited them, but sometimes continue for months, sometimes for years.

The remedies which have been recommended for chorea, may be reduced to four classes, bloodletting, purgatives, antispasmodics, and tonics. Bloodletting, purgatives, and opiates, that is to say antispasmodics, were employed by the celebrated Sydenham,* in a kind of alternate manner, which it is quite unnecessary for me to describe particularly. Purgatives were employed with great success by Dr. Hamilton,† and I may add that I enjoyed the advantage of daily witnessing his practice in this and other diseases, in the Royal Infirmary of Edinburgh. Dr. Currie, whose practice I had the pleasure of witnessing at Guy's Hospital, was very partial to tonics, especially the sulphate of zinc, with which he certainly had great success. No one, I think, has employed antispasmodics alone.

Having tried all these plans, I should recommend you, if the patient be young and vigorous, and especially if he or she have any fixed pain, to draw blood either from the arm, or by means of leeches applied to the part affected. I did so, that is, I applied leeches to the head in the case before you, which immediately removed the pain. I should then, with a view to remove the great accumulation from the intestines, administer one of the pilulæ aloës cum myrrhâ, every hour during waking. It is necessary to give them every hour in order to keep up completely the peristaltic action of the bowels; and you will be completely astonished, if you inspect the feces every day, which you certainly ought to do, to witness the appearance of them. In the first place the quantity of them will surprise you; in the second place, the great varieties of colours will a little amaze you. You will see part of them black, part brown, deep yellow, green, clay-coloured. When you have continued the purging until the feces are of a uniform and natural colour, and you will be surprised to find how well the patients bear this purging, you may then omit the purgative pills, and give a tonic pill of one or two grains of sulphate of zinc, three or four times a day; with a sulphuric acid mixture, containing a proper and regular dose of the acetate of morphine. You may also, at any period of the treatment, employ the shower-bath. The reason why I employed the hot bath in Grace Plant's case, was the fear that her disorder had, in some measure, arisen from the partial suppression of the lepra; and also because the hot bath would at any rate be beneficial to her lepra. If you think that suppression of the catamenia has any thing at all to do with the complaint, you may give the pilula ferri composita; although the constant purging with the pilula aloës cum myrrhâ will most probably produce a very beneficial effect upon the uterine system, in cases of the partial or total suppression of the catamenia.

If you proceed upon the principles just laid down to you, I cannot doubt that you will have the same success in every case of chorea coming under your care, as I myself have had."

* 'Schedula monitoria.' Sect. 20.

† Hamilton, on Purgative Medicines.

### II. Jaundice.

Dr. Carbutt details two cases, and subjoins a few remarks, which, being essentially founded on the former, cannot be justly appreciated without them. We present the cases, in some degree abbreviated.

Case 1.—*Jaundice, from Inflammation of the Duodenum.*

Mary Ann Hollingsworth, æt. 41, unmarried, admitted August 12, 1833.

The skin and conjunctiva are tinged of a deep yellow—pains in the limbs—pain in the right side of the epigastrium, over the duodenum, but none in the region of the liver—much depression of spirits—tongue furred—appetite impaired—bowels purged, and fæces loose and clay-coloured—urine very dark—no catamenia for the last twelve months—pulse 84—sleeps badly, and is troubled with terrifying dreams.

The complaint appeared a fortnight ago with pains in all her limbs, sickness and purging, and yellowness of the skin.

To have twelve leeches applied to the right side of the epigastrium, over the duodenum.

To take one mercurial pill night and morning.

15th. Decidedly less pain in the epigastrium since the application of the leeches. Has still pain in the thighs and legs. Tongue cleaner. Appetite better. Bowels open. Feces bilious. Urine very dark coloured.

To have ten leeches applied to the right side of the epigastrium.

On the 14th, the pain in the side had disappeared. On the 16th, the tongue was clean, and the excretions had become healthy. The pills were discontinued, and the following mixture prescribed.

℞. Potas. carb. ʒij. Tinct. aromat. ʒiv. Infus. calumb. ℥ vss. M. Capt. ℥iss. 4 ter die.

From this time she continued to improve, and, on the 2d of September, was discharged cured.

"I knew, gentlemen," says Dr. Carbutt, "that this case of jaundice depended on inflammation of the duodenum, partly from the pain upon pressure over the duodenum, and partly from the circumstance that the disease was ushered in with sickness and purging, pain in all the limbs, and the sleep disturbed by terrifying dreams. I apprehend the mode in which inflammation of the duodenum produces jaundice, is by the inflammation extending up the ductus communis choledochus, and thickening the mucous membrane so as to choke up the passage. You have seen how rapidly the disease was removed by the application of leeches to the epigastrium.

This species of jaundice is not, as far as I am aware, mentioned by any author. It may, perhaps not inappropriately, be named *icterus duodenalis.*"

The cautious pathologist might not experience the certainty of Dr. Carbutt on the jaundice, in this instance, arising from inflammation of the duodenum. He might hint that the circumstance of pain being felt in the direction of the duodenum, and the symptoms of sickness, diarrhœa, pains in the limbs, and sleep disturbed by terrifying dreams, are insufficient to prove the existence of a new species of jaundice. Those symptoms may be admitted to establish its probability, but rigorous reasoning would admit no more. The practical physician may possibly content himself with the issue of the treatment. It was such as the symptoms seemed to indicate, and whatever was their cause, it effected their removal.

Case 2. *Hepatic Jaundice.*

Anne Charlesworth, æt. 56, married, was admitted June 11th, 1833.

The skin and conjunctiva are deeply tinged with yellow. The hepatic region is not painful on pressure; but she says she is frequently seized with acute pain in the right side, which extends upwards to the right shoulder: when thus affected she can lie with more ease on the right ride. Has some pain in the loins and down the right thigh. Much lassitude and debility. Sleeps well, but has startling dreams. Pulse 80, soft, and regular—tongue white and furred—appetite bad—bowels very loose, and the motions slimy and whitish—urine muddy, with a bilious tinge.

The complaint commenced in December last, with pains in the whole body, and a constant purging of mucus and of blood. She had also intolerable itching, especially when warm in bed. Yellowness of the skin succeeded these symptoms.

Pil. hyd. gr. v. omni nocte.

On the 14th the blue-pill was ordered to be taken twice, instead of once daily. On the 17th, there was little alteration. She suffered from great depression of spirits, and had several times been attacked with feelings of extreme faintness. She had a sense of burning at the epigastrium. Her low spirits prevented her from sleeping.

She was ordered a morphia draught, and a warm bath every night.

On the 20th she was so depressed that she grew impatient of remaining in the dispensary, from which she accordingly departed.

The following are the clinical remarks of Dr. Carbutt.

"You know, gentlemen, there are usually reckoned four species of jaundice, to which adults are subject. 1. Biliary jaundice, in which the ducts are obstructed by a clot of thickened viscid bile. I apprehend the case before us was not a case of this kind, because it is most probable that before the lapse of six months the obstructing clot of bile would have made its exit into the duodenum. 2. Gall-stone jaundice, in which the passage of the bile is impeded by the presence of gall-stones in the biliary ducts, causing great pain in the epigastric region; which pain, in the case before us, did not exist; shewing that this was not the species of jaundice with which our patient was affected. 3. Spasmodic jaundice, in which the flow of bile is obstructed by a spasmodic constriction in the course of the bile-ducts: which, however, soon gives way, leaving the bile-ducts free. Now, as our patient had been ill six months, it is not probable that her complaint had its origin in so temporary a cause. We come, therefore, to the 4th and last species, Hepatic jaundice, in which the course of the bile is obstructed by a derangement of the liver from scirrhous or other indurations. This, I believe, is the disease of our patient, and is decidedly the worst form in which jaundice ever appears. It is frequently the mark of a broken state of health; it rarely appears, as the other species often do, among the young and vigorous; but mostly among those who have lived in a hot climate, or who have led a life of hard drinking.

As for the treatment, I intended to try the effect of small doses of mercury, continued for a considerable length of time, and when the mouth should become affected, to exchange the mercury for small doses of the pilula aloës cum myrrhâ, which, in a manner that I shall explain another time, produces upon the liver the same effect as mercury. I should also most probably have employed the nitro-muriatic bath, which is said to have a remarkably specific effect upon the liver and its secretion; and if I should obtain a patient fit for its employment, I trust I may yet have an opportunity of exhibiting it to you. The warm-bath and the acetate of morphine I employed for the purpose of giving her good nights, and allaying her excessive general restlessness. This last, however—her restlessness I mean,—increased so very much, and made her so importunate in her entreaties to be allowed to go home, that, taking into consideration the very great hopelessness of her recovery, I at last consented to allow her to leave the house.

In commencing the enumeration of the species of jaundice, I made use of the expression, '*usually reckoned;*' because there is another species not usually mentioned by authors, to which I have given the name of icterus duodenalis, and which has its origin in an inflammation of the duodenum. This inflammation induces a greater secretion of bile, and, when the inflammation extends up the bile-ducts, it thickens the mucous membrane so as to present an impediment to the passage of the bile into the intestines, and thus it produces jaundice. This species has not, as far as I am aware, ever been mentioned by authors."

Dr. Carbutt is probably right in his opinion of the irremediable nature of the jaundice in the instance of the patient whose case we detailed last. Her advanced age, the general symptoms, and the duration of the malady, render an unfavourable prognosis unavoidable. Yet still we may observe that the treatment in the infirmary was too brief and too simple to warrant the conviction of the inefficacy of remedies. Leeches and subsequent counter-irritation in the region of the liver, aperients with alkalies, and afterwards mild tonics—and the gentle exhibition of mercurials are means we should employ, before we abandon the case as hopeless. It is possible that these or analogous measures had been adopted and fully tried, prior to the patient's reception in the infirmary. But they have not been alluded to in the clinical report.

### III. Rheumatism.

This disease has been ranked amongst the opprobria of our art. Yet we think that the accusation is not strictly just, for science can do much to mitigate and cure acute rheumatism, and more to avert those dangerous complications and effects—inflammation of the pericardium, the pleura, and the cerebrum.

Dr. Carbutt considers, under the name of rheumatism, five different affections, and appears inclined to include as a sixth, the tic douloureux. He goes beyond the popular nosology, which allows but three, and which gives to them the classic names of "rheumatism," "rheumatics," and the "rheumatiz." The superficial examination of the vulgar has failed to discriminate those nicer shades distinguished by the eye of the clinical physician.

"The first variety," says Dr. Carbutt, appears to consist in a peculiar, specific, inflammation of the muscular fibres, or of the cellular membrane, or of the muscular aponeuroses, of one or more parts of the body. It is accompanied with much fever, excessive pain upon motion, a tongue covered with a thick, white or buffy coat; a moist, clammy skin; urine high-coloured and depositing a rose-coloured sediment; a full, round, and bounding pulse: the blood drawn is coriaceous, and, for the most part, what is called cupped.

This kind of rheumatism, which is commonly known by the name of rheumatic fever, should be treated by means of copious and repeated bleedings.—Large doses of calomel should be given, first as a purgative, next in order to affect the system. Purgatives, salts and senna, for instance, should then be administered. Diaphoretics and sedatives, the best of which is the pulvis ipecacuanhæ compositus, or Dover's powder, should follow the purgatives. Lastly, large doses of the cinchona, or Peruvian bark, or otherwise of its preparation, the sulphate of quinine, may be exhibited. Low diet must be observed throughout.

The second way in which what is usually styled rheumatism appears, is in the form of arteritis or phlebitis, inflammation of the arteries or veins. I must candidly admit that I know of no precise diagnostic symptoms whereby to distinguish this affection from the rheumatic fever just described. Perhaps, however, the pain is greater and more circumscribed, whilst, at the same time, the febrile symptoms are less intense. This kind of rheumatism should be treated with bleedings from the arm, local bleedings by means of leeches, calomel, salts and senna, Dover's powder in large doses, with low diet.

The third form in which this disease appears, is an acute, hot, and highly painful affection of the joints, as the elbows, the wrists, the knees, the ancles, accompanied with a moderate degree of fever. This is generally called acute rheumatism, it must be treated by general bleedings, local bleedings with leeches frequently repeated, calomel, purgatives, Dover's powder, the hot-bath, cinchona, bark, or sulphate of quinine, low diet at first, generous diet afterwards. The colchicum autumnale or meadow saffron is very much recommended in this affection, but I have no great faith in its effects."

We hesitate to accede to the division drawn by Dr. Carbutt between rheumatic fever and acute rheumatism.—Such divisions are clear and satisfactory on paper, perhaps they may adorn a clinical lecture, but they lose their substantial reality and distinctness in the sick room and the ward. It is true that we find rheumatic inflammation affect in some instances the cellular and fibrous tissues, and perhaps the muscles, in preference to the synovial membranes—whilst in others the latter textures are principally implicated. This fact gives an air of plausibility and correctness to the distinctions which an able physician has drawn between synovial and fibrous rheumatism. It has even been believed with some semblance of truth, that the latter is the form which evinces an exclusive disposition to metastasis. The experienced physician is, however, aware that these doctrines are no more than plausible, that Nature resists the arbitrary law, that fibrous and synovial rheumatism are frequently combined in the same individual, and that affections of important organs, especially the pleura and the pericardium, may occur in both.

Dr. Carbutt asserts that the synovial rheumatism is attended with less fever than the fibrous. As a general rule this may be true, but it is far from being universally the case. In some of the very worst cases of "rheumatic fever" which we have had occasion to observe, the synovial membranes were the parts affected. We would not, therefore, state to pupils in the confident tone adopted by our author, that the two affections are separate and distinct, that one is a more acute variety than the other, and that a different treatment is required for both. Those pupils finding that the lessons of their teacher are not borne out by bed-side observation, and discovering that what was so simple in the lecture-room becomes contradictory and confused in practice, are frequently tempted in despair and disgust to dispute authority, undervalue learning, and trust to routine and empirical experience. Instead of laying down the positive divisions of Dr. Carbutt, we should rather proceed with more caution and more accuracy to state that rheumatism sometimes affects in preference one tissue, sometimes the other, and sometimes, though not so frequently, both—that usually the fibrous rheumatism is a more acute disorder than the synovial—that generally it evinces a more decided disposition to attack internal organs, especially the pleura and the pericardium—and that, commonly, certain remedies are more useful in the one form than in the other. When taught with such philosophical caution, we might almost call it doubt, the pupil will observe with corresponding care, and the apparent contradictions of nature will not astonish and may not perplex.

That division of rheumatism which is seated in the arteries and veins is hinted rather than described by Dr. Carbutt. We must own that we never saw an instance of the kind, and probably neither Dr. Carbutt nor ourselves would recognize it were it to occur. It is doubtful if, under such uncertainty, it is proper to admit the existence of the disease as one of a substantive character. The arteries or the veins may inflame in rheumatic cases, as the pleura, the pericardium, the mucous membrane of the bowels, perhaps every texture in the body may do. Yet the present condition of our knowledge would lead us to deem them consequences or complications rather than primary rheumatic symptoms.

It is curious that, although our author alludes to this obscure and dubious affection of the blood-vessels, he omits to notice the frequent and formidable affection of the pericardium. Yet, if any great improvement has been effected with respect to the management of rheumatism, it arises from the attention which has been directed to the heart. The experienced practitioner anxiously watches and promptly meets any symptoms of pericarditis and of pleurisy; he knows that they are common, insidious and formidable.

We lately saw an instance of fatal affection of the mucous membrane of the bowels. A girl, aged eighteen, had, twice or thrice previously, suffered from acute rheumatism, of the mixed fibrous and synovial character. In each attack, there had been symptoms of some implication of the pericardium. This Winter she was again affected in a similar way, and was apparently recovering, when, at mid-day, she was seized with bilious vomiting, purging, and pain in the region of the colon, rapidly succeeded by collapse. Soon after midnight she died. Some little time ago, we witnessed a case of the same description. Blood was voided in the stools, and for some time the patient was in the utmost danger; but he did not die.

Dr. Carbutt, it would seem, is an advocate for copious and repeated bleedings in rheumatic fever. We doubt the propriety of this practice. It does not cut short the malady, but it greatly debilitates the patient, and we are not sure that it does not tend to favour the occurrence of metastasis. At all events, patients go through the disease well without copious bleeding, and we seldom or never have recourse to it. Of course we are speaking of an ordinary case, and of the ordinary treatment.*

"The fourth form of rheumatism is called chronic rheumatism, and is exactly the same as the third, except that all its symptoms are chronic, and there is little or no fever. It must be treated by leeches repeatedly applied to the painful joints, hot baths, blisters, calomel, stimulating liniments, cinchona-bark, ammonia internally, generous diet, the affusion of cold water on the painful joints, or lastly, frequent bathing in the Buxton baths.

The last is an almost never-failing remedy; and, if I am asked how it operates, I answer, I do not know. Some substance, as yet undiscovered by chemistry, exists, as I conjecture, in Buxton-water, on which probably its efficacy depends. The beneficial effects of Cheltenham-water in enlargements of the liver and spleen were acknowledged many years before the very existence of iodine in nature was known. Now, it is understood that iodine exists in Cheltenham-water, in the proportion of one grain in ten gallons. 'It seems not improbable,' says Dr. Daubeny, Professor of Chemistry at Oxford, 'that very minute portions of certain principles may act upon the system with an energy commensurate, not to their own quantity, but to the change their presence occasions in the properties of the more inert ingredients that accompany them.—In this manner we may explain the powerfully tonic effects of certain springs containing a very minute impregnation of iron; the cures effected by waters, such as those of Loueche or Gastein, which appear to approach as nearly as possible to absolute purity; and the efficacy in glandular disorders attributed to certain others, in which a minute proportion of iodine or bromine has been detected. In a Memoir read before the Royal Society, on the saline and purgative springs of this country, in which I stated the proportions of iodine and bromine present in each, I expressed myself as being sceptical with regard to any medicinal agency that could be exerted by so mall a quantity as one grain of iodine diffused through ten gallons of water, the largest quantity in which I had ever detected it. ' The considerations above stated now induce me to attach more importance to the circumstance of its presence, for it is just as possible, *à priori*, that this quantity of iodine should infuse new properties into the salts which accompany it, and cause them to act in a different manner upon the system, as that less than a millionth part of potassium should create so entire a change in the relations of a mass of mercury to electricity. Whether the waters of Cheltenham or Leamington affect the constitution differently from the solution of Glauber salt, of similar strength, must be decided by the experience of those on the spot; but, granting this to be the case, and there is not wanting testimony in favour of such an opinion, the discovery of these new principles in several of them may serve to explain their superiority.'—I leave it to you, gentlemen, to decide, each in his own mind, how far these considerations apply to the undoubted efficacy of the Buxton-baths in cases of chronic rheumatism."

By the way, there are two remedies to which Dr. Carbutt makes no allusion—guaiacum for acute rheumatism, and mezereon for the chronic. Yet the mistura guaiaci is a very useful and a safe remedy for acute synovial rheumatism, and we have really seen it answer in a remarkable degree. It produces extreme diaphoresis.

The fifth form to which Dr. Carbutt adverts is sciatica.

"This affection (he remarks) sometimes attacks other parts besides the hip, as the face, the foot, and the mamma. It appears to me, that it is essentially an inflammation of the membrane which invests the nerve of the part affected. This is shewn by the intense pain, the sudden paralysis, so that a person walking in the street shall fall down as if in a fit, from which cir-

* In the following number of the Journal, Dr. Johnson means to attempt a distinction between what is called acute and chronic rheumatism—or rather to dislocate them. The former he would designate arthritis—the latter rheumatism.

cumstance, no doubt, it has received the name of paralytic rheumatism, or rheumatic paralysis. The coldness, numbness, and wasting of the muscles, with the almost total absence of fever, altogether lead to the opinion, that it is more an affection of the nerves than of any other parts. When it exists in the face, it very much resembles the *tic douloureux;* or, for any thing I know to the contrary, the tic douloureux may be the same disease; which I think extremely likely. It should be treated, I apprehend, by means of local bleedings by cupping-glasses or leeches, by blisters, by mercury, especially calomel, so as to affect the mouth, by sedatives, as opium, extract of hyosciamus, or Dover's powder. A very good combination is, one grain of calomel and five grains of extract of hyosciamus, four times a day. Usually, as soon as the mouth is affected with the mercury, or even sooner, the disease yields as if by a miracle."

We have, on several occasions, rapidly cured old sciatica in this manner. We have ordered the patient to take five grains of calomel, a grain of opium, and half a grain of the extract *of* the acetum colchici, for four or five nights successively, adding, on the following mornings, a very active purgative of senna and colchicum, &c. We have twice or thrice had the patient cupped over the sciatic nerve, and then applied blisters in the same situation. These means combined together, and conjoined with the warm bath, have, in several instances, speedily routed the disease, which had resisted, perhaps, the same measures employed in a more desultory manner.

"There are two affections which it is my duty to caution you against mistaking for rheumatism. The first is that inflammation of the periosteum which we find in secondary syphilis, and which gives rise to those syphilitic pains which, as you must all know, are so frequently mistaken for rheumatism. The second affection is that pain of the shoulders and arms, of the back and loins, of the hips, thighs, knees, legs, feet, and toes, which I have mentioned in former lectures, as being symptomatic of gastro-enteritis, and as being very often mistaken by even elderly practitioners, and as regularly treated by them, for rheumatism, to the great injury of the patients, and the aggravation of their real complaints."

Dr. Carbutt relates nine cases, six of which were chronic. The exposition of his doctrine renders a notice of these cases unnecessary, especially as they display no peculiar features of importance.

### IV. Amenorrhœa.

Three cases of this disorder are related. In one the cause was pregnancy, which the lady at first concealed, a common occurrence in hospital practice. In the other two instances the amenorrhœa was of a more orthodox description. We notice them in consequence of digitalis having been employed.

*Case* 1. Betsy Linney, æt 20, a factory girl, of strumous habit, and of pale complexion, had never menstruated.—She was much troubled with wind and globus hystericus. Pain in the epigastrium increased after eating, but not increased by pressure. Pain in the back, shoulders, and calves of the legs. Has some ulcerations on her back.—Pulse rapid and small. Tongue streaked with white and slightly red at the tip. Bowels very irregular, sometimes costive, at other times very much relaxed.

Leeches to the epigastrium, a sulphuric acid mixture, rice diet, and castor oil, were the measures directed against the symptoms of gastro-enteritis. Those symptoms were relieved, and the girl was then ordered to take fifteen drops of the tincture of digitalis four times daily in the acid mixture. In six days after commencing this medicine, she felt very sick and faint, and the pulse was slow and regular. The digitalis was omitted, and on the next day she went out at her own request.

Dr. Carbutt observes, that as no very serious complications existed in the preceding case, as no important organ exhibited important alterations, he thought that a fair opportunity was presented for testing the effects of digitalis on the catamenia.

"Digitalis is said to have a peculiar determination to the genital organs, both in the male and in the female; so much so in the latter, as to be capable of producing abortion. However our patient took it for seven days only, when it produced so much sickness and fainting, and such a lowering of the pulse as to oblige me to omit it. It had produced no effect on the genital organs that I know of. Had the patient continued in the house, I should most likely have resumed it in a few days. When this patient came into the house she evidently laboured under inflammation of the stomach and intestines, which, however, was quite unconnected with her amenorrhœa. For this I treated her by leeches, poultices, and a mucilaginous diet. She was also of a very strumous habit, which was my reason for giving her the sulphuric acid mixture. Had she remained in the house, and the digitalis had entirely failed in bringing on the catamenial discharge, I should have administered the 'pilula aloës cum myrrhâ,' or the 'pilula aloës cum ferro,' or the 'pilula ferri composita,' or the 'mistura ferri composita;' all of which undoubtedly possess very great efficacy in this respect, and may be confidently recommended to your employment; as, if there be such things as emmenagogues, these are certainly they."

It appears to us that Dr. Carbutt's observations are extremely incomplete. An emmenagogue is, after all, a relative remedy. Menstruation may be stopped by debility, by the agency of cold, by the existence of disease in some other organ than the uterus, by mechanical obstructions, and, in short, by many causes. The same remedy will not be applicable in all these instances—will not, in short, be an emmenagogue. If there be debility, tonics are emmenagogues—if constipation, purgatives *may* be such—if another malady exists, its removal is an emmenagogue—in short, the emmenagogue, in many cases, is simply the correction of what is wrong in the system, or a part. Yet some medicines exist which exert, under many and different circumstances, a peculiar action on the uterus—such are the savine and the ergot of rye. The judicious practitioner is he who is able to distinguish the cases for general and for special remedies. What madness it would be to prescribe the steel, or galbanum, or savine, in a case of amenorrhœa, attended with plethora, with a foul tongue and a full pulse. Yet such madness is not unfrequently observed; and the pupil who is told that such remedies are emmenagogues, without being informed of the various causes and characters of amenorrhœa, without being instructed that what is useful for one case is injurious for another, is apt, we should imagine, to be such a lunatic as we have hinted at.

*Case* 2. Maria Grant, æt. 17, admitted August 15th, 1833. Is a piecer in a cotton-factory, says she has not menstruated since last Whitsuntide, the last week in May, and has, from that time, felt pain in the stomach and bowels, not constant but at times, particularly towards night, very acute. Bowels rather loose. Tongue furred at the centre, red at the sides. Bad taste. Sleeps ill. Appetite bad. Pulse 98. No cough.

℞. Tincturæ Digitalis f℈j.

Misturæ Camphoræ f℥vj. Misce.

Sumatur f℥jss. quater quotidie.

Pain in the stomach, aggravated after meals, with whitish tongue, and, perhaps, a little acceleration of pulse, followed hard upon this treatment. The digitalis was, in consequence, omitted, and leeches were applied to the epigastrium. The gastro-enteritic symtoms were relieved, and the pilula ferri composita was given. On the 2d Sept. the patient is said to have been discharged cured, but no mention is made of the re-establishment of the catamenia.

The digitalis obviously did no good in this instance. The general treatment adapted to the symptoms was productive of most benefit.

We shall return to this volume on future occasions.

---

NEW WESTMINSTER HOSPITAL.

This hospital, situated immediately opposite Westminster Abbey, is built for the reception of 240 patients, and is a very handsome structure, in the Elizabethian style of architecture, rendering it in good keeping with the Abbey. Ten wards were opened the first week in November, and several very interesting cases have occurred since. Sir A. Carlisle has given one clinical lecture on the management of burns and scalds generally, and Mr. Guthrie two with reference to particular injuries in the hospital, of which the following is a condensed summary.

Mr. Guthrie first expressed his satisfaction at having a case which would complete the picture of maniacal symptoms supervening on injuries of the head, which he had drawn to them a few evenings before, when on those injuries in his general lecture, and which he had said was rare. In this case the man, Samuel Charles Deacon, aged 37, admitted 17th November, at night, having fallen from a height on his forehead, which was much bruised about the right frontal eminence, although no fracture could be distinguished he was a good deal stupefied on being brought into the hospital, but was capable of being roused when spoken to sharply. He was bled to 16 ounces from the arm and had a cathartic dose; which was repeated until the effect was produced. He was bled again to the same extent, and the purgative medicine was repeated from time to time. The second blood drawn was buffed, pulse 100, soft and regular. Pain in the head slight: ordered to be shaved and cold applied. He lies in a state of indifference to all around him, but returns an answer when roused: mutters in his sleep at night.

On the 21st at night he began to be restless, wandering, and noisy, so that at last he was obliged to have a straight-waistcoat put on—discharged his fæces involuntarily—pulse frequent but soft—pupils sensible to light, and not dilated—a blister was applied between the shoulders. I saw him on the 22d, and at first sight said that is one of the very cases I have been speaking to you about. This is not phrenitis, it is maniacal delirium, and depletion will do harm, the loss of 16 ounces of blood will probably kill him, whilst an opposite treatment may be effectual. He was sitting up in bed in the straight-waistcoat, and trying to get out of it, talking very incoherently and unconnectedly, but seems to be able to attend and to reply when spoken to sharply. The pulse quite regular, soft, and without power. I directed half a grain of the muriate of morphia to be taken immediately, and a grain to be administered at nine at night, after which he became more tranquil, and slept, although at intervals he was noisy. The next morning, the 23d, he was much better, the pulse being but 80 and fuller. He was purged, and the cold lotion applied to his head, and the morphia was repeated at night. The purgative and quieting treatment were continued until the 2d December, when he was able to sit up and walk about, and appears quite natural, and says he is free from pain in the head. On enquiring it was found that he was thought more or less silly by his friends, although he had never been actually out of his mind; and I believe in these cases there is almost, if not always, a tendency to mania in the habit. Some time ago I saw a case of a similar nature in this hospital. The man was bled largely instead of having purgatives and narcotics, and died, but on examination no signs of inflammation could be discovered in the brain or its membranes. This case is a remarkably good practical one, and should be strongly impressed on your minds, for if you mistake the maniacal derangement for the delirium accompanying phrenitis, the error will be probably fatal. The peculiar vacant maniacal look in this case was very distinct, and as far as I have been able to observe it is usually so.

The case of William Isaacs, who lay in the next bed, under the care of Mr. Lynn, is not less interesting, as exemplifying many of the facts I have stated to you in the general lectures also. This man was run over by a cab on the evening of the 24th November, and died on the 3d December. The pulse during the greatest part of his treatment ranged from 94 to 100, and was full and regular, yet there was a considerable effusion of blood on the brain from the middle meningeal artery, which had given way on the inside of the dura mater, at which a large clot had formed, independently of that which was otherwise exposed towards and on the base of the cranium. As death approached, the pulse became very rapid and very small. According to the older, and even some modern surgeons, the pulse should have been slow, soft, and weak, if not irregular. Theory says it ought to be so, when there is compression of the brain, and in theory it may be true; but I tell you that, in practice, you will more often find it the reverse, and that the pulse often shews nothing, gives no diagnostic sign of the nature of the injury received. You have in this case the proof. The pupils of the eyes were never dilated, at least up to the last time I saw him, on the morning of his death. They were certainly not active under the influence of light, but they were fully sensible to it; yet there was compression of the brain to a considerable extent. I therefore tell you, that a dilated and inactive state of the pupils depends, not upon compression alone, but on compression on *a particular part* of the brain, and that part is not on the side of the middle lobe, or on the under part of it, or of the anterior lobe.

This man suffered exceedingly from shiverings and convulsions, which latter were very severe, and I ventured to predict a laceration of the substance of the brain prior to the post-mortem examination; and this was found on the left anterior lobe, resembling more, however, a softening and bruising of the part, than a clean tear or laceration, and which is always the case. The appearance is, however, very distinct, and will always be known after it has been once seen. This man's limbs were very paralytic, and he was never sensible, although he occasionally seemed to understand what was said to him. It was a case, also, of great interest, and merited your best attention.

There was no mark externally, or sign of injury, sufficient to warrant the application of a trephine; yet, if one had been applied just over the lower and inferior angle of the parietal bone, the extravasated blood would still not have been seen; but there would have been a sign to tell you it was there. It would have been the absence of the pulsation which is observed on the surface of the dura mater, when the parts beneath are sound, from the action of the arteries of the brain, and the dura mater, instead of remaining on a level, would have risen up more or less into the opening made by the trephine. This indicates the effusion below, whether of blood or of matter, and calls for an opening being made into it, for the evacuation of the blood or pus which may be beneath. I have done this in some instances with success. The result is more usually fatal.

In the bed opposite to these two men there is another, whose case is not less instructive. The poor man, William Holmes, aged 68, fell 23 feet on the ground, on the 23d Oct.; dislocated the left shoulder, and suffered a compound fracture of the ulna, separating it from the olecranon process. The wound enabled me to see the separated parts, and to ascertain that the cavity of the joint was opened. The inflammation was very severe, and the swelling of the whole arm considerable. Matter formed in various directions, and several incisions were necessary, some of them communicating with the joint. A great deal of constitutional irritation took place of course; he has also been much troubled by diarrhœa, and now, at the end of seven weeks, the external wounds are healing, the swelling is nearly reduced, and there is motion in the joint. The fractured ends of the bone are not quite in apposition, and direct union cannot immediately take place. I have hitherto kept the arm nearly straight, or as straight as it can be kept without bandages; but, as I see consolidation going on, and the prospect of the arm being about to become stiff or anchylosed, I shall place it in a semi-flexed or half-bent position, as it will be then more useful. The case has altogether been well worthy your attention; but my principal object in addressing you is with reference to the two positions. All the injuries of the elbow-joint require the bent position save one, and that a fracture of the ulna, at or near its olecranon process; and a mistake as to the nature of this injury is very distressing to the patient. A gentleman applied to me, some years ago, with the view of obtaining relief under the following circumstances. He had fallen on the under part of his right arm, which swelled exceedingly, and was treated by placing the arm in the half-bent position until it had entirely subsided, and all pain was removed. On attempting to use it, he found he could not straighten it by any muscular effort depending on volition, save that of applying the other hand to it, when it was effected with ease. If, for instance, he took up a piece of bread, and put it into his mouth, he could not bring his hand away by its own muscular efforts, but was obliged to put it down with the other hand. If he attempted to shave, he could only apply the razor to his face—all further motions he could not effect. The appearance which this defect gave rise to was very ridiculous, and not less disagreeable. On examination, I found that the ulna had been broken, immediately below the olecranon process, and that union had not taken place, either by bone or ligament, so that, on bending the arm, the separation between the parts, to the extent of half-an inch, could be distinctly felt, the end of the shaft of the ulna being apparently quite smooth. He had thus lost the use of the triceps muscle, and I, for the first time, found out all its many points, connected with its services, with which I was not acquainted. Few teachers of anatomy, I believe, describe among them, its being the principal muscle used in shaving; and, although they laud the size of the biceps in a blacksmith, I am not aware they praise that of the triceps in the barber. I told him the only thing to be done was, to make an incision over the end of the ulna, then scrape the ends of the bones, and bring them firmly in apposition, in as straight a line as possible. I did not, however, conceal the danger which might arise from inflammation of the joint, and I believe the gentleman did nothing, beyond tying a spring, fitted on his arm behind, the effect of which I did not learn.

I have lately seen another gentleman, with an accident something resembling this. He was thrown out of his gig on his elbow, and broke the ulna joint below the smooth place, beneath the insertion of the triceps, and nearly at its apex, so as to make a pointed triangular end to the olecranon; the arm was bent, and, after the lapse of several weeks, I found that the union was incomplete, being effected by an extensible ligament, so that, when the fore-arm was forcibly bent, this pointed piece of bone appeared ready to force its way through the integuments. When the fore-arm was forcibly extended, the broken bones were brought into apposition. The error, in this case, consisted in bending the arm at the commencement of the treatment, and which was rather stiff in that position. This gentleman's anxiety was directed to the recovery of the bending power, or the removal of the stiffness, about which there could be no apprehension; mine was to effect a good union of the bones, and, after giving them some motion on each other, I recommended the arm being steadily extended for a time, with the hope that a better union might take place. The point of bone will, I have no doubt, be very much rounded off, so as to leave little fear of its protruding except from a blow, and the ligamentous union may suffice to give a fair degree of the use of the triceps. If not, the same sort of operation, only in a much less degree, will be necessary, and there will be little fear of mischief in the joint. In all these cases, the defect is not likely to be in bending the arm; a steady use of passive motion, duly applied, will always recover it. The straightening of the arm by the will of the patient is another matter. [Mr. Guthrie here explained the motions of these parts on the bones.]

When the point of the olecranon process is broken off just where the triceps is inserted, little or no displacement takes place, the insertion of the muscle forming a sort of hinge, which keeps the parts together, to say nothing of the ligamentous structures of the joint, which, on other occasions, or a little lower down, principally effect this object. This injury is not always easily detected, unless the surgeon has once seen it, and then it is made evident by bending the fore-arm as much as the patient can permit. In such a case, every motion of the arm and joint can be effected by the surgeon in a satisfactory manner. Yet he occasionally, in going through them, feels a crepitus, although very indistinctly. If, under these circumstances, he now bends the fore-arm on the arm, and places his finger on the upper end of the olecranon process, he will feel the crepitus under it, and ascertain the injury. There may be even a little displacement or separation, which renders the case quite clear. I did not understand the first case of this kind I met with, now many years ago, and kept the arm bent during the treatment, and my patient never could straighten it effectually afterwards. The second patient I met with was the articled student of a surgeon of eminence in this town, who had mistaken the case, as I had done before him. I was in time to point it out, and to effect a perfect cure. Sir Astley Cooper has since published on this particular kind of injury, and which is, therefore, well known to the profession at large.

In all cases of doubt, bend the arm; but as this kind of case, or the cases I have described, ought not to be cases of doubt, straighten the arm. It is easier to bend it at the end of a week or two, than it is to straighten it.

---

### Pennsylvania Hospital.

A long and minute report of eight cases that occurred at this institution is presented in our contemporary, the American Journal of the Medical Sciences, for November, 1834. The cases are possessed of interest, but the minuteness of detail is perhaps excessive, and the crowd of unimportant, if not impertinent, particulars serve rather to annoy than to instruct.

Reporters too frequently commit a capital mistake. They imagine that what is right on the part of the observer is also right on the part of the narrator—that, as a careful surgeon watches symptoms closely, a good reporter should state them as minutely. Yet a little consideration would show the absurdity of this idea. Observation is necessary in order to elicit truth, and unless that observation descends to small things, error may ensue. But he who wishes to inform others, is not to expose the whole process of reasoning by which he obtained the information he conveys. So, in a report, if every circumstance that was noted is recorded, if the reader is informed, not only of all essential circumstances, but of unessential particulars too, his attention is overpowered by fatigue, and his mind is distracted by the multitude of petty objects that surround him. With this remark, we proceed to some of the cases before us. Five are instances of injury of the head. The first presents no feature to attract us.

Case.—*Compound Fracture of the Skull, with Depression—Recovery.*

A labourer, æt. 30, admitted April 3d, 1834.

"While engaged in blasting rocks, a few hours before, he was injured by an explosion; he was alone, and is unable to state the cause of the accident, or attending circumstances; he was stunned, and, on recovering, walked nearly half a mile before he received assistance; he also states that, after he became sensible, was chilly and had vomiting.

A large portion of the scalp was turned off, and a portion of scull, about one and a half inches square, denuded of pericranium, on the inferior and middle part of right parietal bone; a fracture was discovered at this part, one inch in extent, into which a piece of leather, apparently a part of the lining of his hat, had been driven: there was also a small portion of the bone slightly depressed, circular, and nearly one-fourth of an inch in diameter, and a cut in the forehead, extending to the bone. The leather was removed from fissure, and, as he complained of severe pain, Tr. opii, gtt. xl. was administered; wounded parts of scalp kept in apposition by simple dressings."

A purge was given on the following day, and, on the 5th, he had slight fever, with but little headache. He was ordered to take a solution of emetic tartar. On the evening of the 6th, he became restless, complained of pain in the head, and had fever. He was bled to ℥xv. which caused faintness. The solution of antimony, producing vomiting, was discontinued. On the next day he was better, and was ordered merely salines. On the 12th, there was an abundant purulent discharge from the scalp. He went on well till the 25th, when he suffered from headache, some augmented frequency of pulse, and inability to sleep. The wounds of the scalp had healed, excepting over the exposed bone. A blister was applied to the back of the neck, and purgatives were subsequently given. The symptoms were relieved. On the 31st of May, the exposed portion of cranium, which was previously loose, was removed by cutting down upon the parts; the dura mater was healthy. The patient had occasionally slight headache, which a brisk purgative commonly removed. On the 31st of June, he was dismissed, cured.

This case exhibits an example of the fact, that compound fracture of the cranium with depression, though dangerous, is sometimes not succeeded by severe symptoms. The depression was certainly slight, the injury to the cerebrum was probably trivial, for the patient was affected with the symptoms of concussion rather than compression. The injunction to trephine, in *all* cases of compound fracture of the cranium, with depression, may at times be disregarded.

CASE.—*Compound Fracture of the Skull, with Depression—Convulsions—Death.*

A labourer, æt. 47, admitted April 23d, 1834, at 7, p.m.

"Is of intemperate habits, has generally enjoyed good health; was struck on the head by the crank of a crane, at which he was employed hoisting logs, about one hour previous to his admission into the hospital. The blow was received on the right side of the head, at the anterior inferior part of the os frontis, immediately over the orbit of the eye; was insensible for a few minutes after the accident; had no vomiting. When admitted, pulse was feeble and frequent; skin cool; rationality good; has a cut four and a half inches in length in scalp, which is turned off, exposing a considerable portion of the cranium, partly denuded of periosteum, a fracture by which the upper part of the orbit was forced down over the eye; a small loose fragment was removed; there is also a cut half an inch long at external canthus, from which there is slight hæmorrhage; eye uninjured. The depressed portion of bone was elevated to nearly its natural position, and light dressings applied; patient complained of no pain, and rested well during the night."

Nothing of consequence occurred till the morning of the 25th, when there were chilliness and increased restlessness; the pulse was feeble. At half-past one, p.m. the patient suddenly became convulsed and comatose, with pulse full and frequent; pupil nearly natural; strong flexion of arm on uninjured side during the attack. He was bled to ℥xx. and had cups applied to back of head, after which he became sensible. The articulation was now indistinct—the pupils were slightly dilated—the left eye was drawn strongly to the internal canthus—pulse frequent and weak—skin warm. At 11, p.m. the pulse was irregular—the respiration slightly stertorous—the left eye was fixed at the internal canthus—he was very restless. At 9, a.m. of the 26th, low, muttering delirium; increased restlessness; eyes as last reported, with more dilatation of pupil, and complete insensibility to light; surface of body, natural temperature. The separated fragments of bone were this morning removed, requiring only detachment from a small portion of scalp. He sank rapidly, and died at 4, p. m.

*Dissection*. The wound was of some size, extending from near the inner canthus of the right eye upwards and outwards for four inches and a half, and exposing the frontal bone for the distance of three inches. Dura mater opposite the seat of fracture, covered with a thick layer of dark coagulated blood, adhering strongly to the membrane, in the midst of which were two perforations, the largest of which would admit a crow quill. The dura mater and the arachnoid were each injected. Above and below the arachnoid of the pia mater, was a purulent cream-like fluid. The cavity of the arachnoid contained posteriorly half-an-ounce of dark fluid blood. The pia mater and contiguous brain were injected, and some ecchymosed spots were remarked in the cortical substance of the latter, those ecchymosed portions being softened in the centre.

The patient would appear to have suffered and died from the inflammation set up about the injured parts, rather than the amount of immediate injury. Yet the consequences of the inflammation, suppuration, and softening, were inconsiderable, and disproportioned to the rapid fatality of the case. The practical surgeon has frequent opportunities of observing instances of a similar description. It is probable that the circumstance of one individual being killed by a smaller amount of disorganization than is capable of terminating the existence of another, depends on their relative constitutional power.

The supervention of convulsions in connexion with the symptoms of commencing pyrexia, is interesting and was in all probability dependent on inflammatory action. Moderate pressure from extravasation or depressed bone will produce convulsions; but then febrile action is not present.

CASE. *Fracture of the Skull—Phrenitis—Death.*

A boy, æt. 12, was playing on the roof of a three-storied house, from which he fell, on the afternoon of the 10th of August, 1834. At his entrance into the hospital, had a large ecchymosis with tumefaction of the eyelids of the right eye and parts covering the malar bone and temporal fossa; a fracture existed in both bones of both forearms, about two inches from the wrist. Intellect clear, but much agitation, and complained of pain in the arms, which were dressed by the application of splints and compress. Until 15th, slight increase of pulse and heat of skin; great restlessness; complained of pain in arms, constantly throwing them about; intelligence perfect, but very irritable, and great sensibility to impressions.

"On 15th, after sleeping quietly, ($\frac{3}{8}$ths gr. of morphia having been given during preceding day,) had great increase of heat, no chill remarked, but extreme stupor, lying without the least attention to surrounding impressions. At 8, p. m., found him in the following state: decubitus dorsal; eyes closed; arms lying by his side; not much tossing about; skin intensely hot; face flushed; pupils much and equally dilated, little contraction on exposure to light; sensibility generally augmented; cries on moving the limbs; sight appears nearly perfect; hearing good; quickly relapses into the state of stupor when aroused; pulse 140, quick, regular; respiration regular, 28 to 30; no cough; constipation.

℞. Iced water to head; Pulv. seidlitz, No. ij.

16th. Very restless during night; frequent cries as if in pain, alternating with stupor; some dilatation of pupils, and increase of sensibility; pulse 140, quick; head strongly bent backwards; cries if attempts are made to replace it; thirst great; no requests *for food;* face flushed; no rigidity; violent delirium, or during night cries; gives intelligible answers to questions if loud, and then relapses into a state of coma. VS. ℥xviij. After bleeding face pale; less violent delirium, pulse much more feeble.

In evening pulse less feeble than in morning, some delirium; skin again hot. Twenty leeches behind ears: blister over scalp.

During the three days, 17th, 18th, and 19th, the following symptoms were observed. Delirium, quick, irregular, and noisy, with sometimes cries. On 19th, coma; pupils dilated during whole attack, not sensible to light on 18th or 19th. Strabismus, 17th, 18th, and 19th. The inclination of the head backwards increased in strength, but there was no evident rigidity of the limbs; no distortion of the features, unless slightly puffing of the mouth; countenance anxious, flushed; no replies to questions after 18th; constipation relieved by enemata or Seidlitz powders; no vomiting.—Deglutition impossible after 18th; meteorism of abdomen on 18th and 19th. Complained of pain in belly on 18th; pulse regular, very frequent, from 120 to 180.

Death 20th, at 9, a. m."

We have given the preceding case at length, because it is interesting to trace its progress, and not uninstructive to discuss its treatment. Let us glance at both.

A boy receives a severe fall upon the head, apparently unattended with the symptoms of concussion or compression; he has also fracture of bones of either forearm. For five days after the accident he has pyrexia, excessive irritability, and great sensibility to impressions. For these symptoms morphia is given, and, after it, he falls into a state of stupor, with dilated pupils, flushed face, hot skin, and pulse at 140. For all this iced water is applied to the head, and he takes a Seidlitz powder. The symptoms increase—delirium alternates with coma—the head is bent backwards as in opisthotonos—the fever is decided. He is bled with some relief. The coma gradually grows more complete—strabismus is established—the inclination backwards of the head is aggravated—deglutition becomes impossible—and in ten days after the accident the patient dies.

The experienced surgeon cannot fail to recognize in this march of symptoms the characters and progress of inflammation of the brain or of its membranes. The *experienced* surgeon would also have acted, or probably he would have acted with more boldness and more judgment than were actually displayed.—Five days elapsed after the occurrence of the accident, an accident well-known to be likely to occasion inflammation of the brain; yet, although there were fever, irritability, and morbid sensibility to impressions, no depletory treatment of any consequence was instituted. On the contrary, morphia was exhibited, a medicine which should ever be carefully avoided, or most cautiously employed for symptoms succeeding to injuries of the head. There is much in this that we cannot praise, much that a critical judgment might condemn. But more remains behind. A state of stupor, with dilated pupils, and decided fever, succeeded the employment of the morphia. Again, the experienced surgeon, perhaps even the well-instructed student, would have feared phrenitis, would have used depletion. The depletion that *was* used consisted in a Seidlitz draught.

The nature of the symptoms and inertness of the treatment might naturally lead the pathologist to look for inflammation in the cranium, and its consequences—lymph, or purulent effusion. The strabismus and retraction of the head might induce him to suspect mischief at the basis of the brain. Let us pass to the examination of the body.

*Dissection.* "Dura mater distended; long coagulum in sinus; fracture with depression of both tables of os frontis on right side, just above the external angle of orbit; fracture extends through the orbitar plate in its whole breadth; just above this fracture the dura mater is torn, and a coagulum of black blood an inch in breadth exists. Summit of convolutions compressed; arachnoid dry; no serosity in pia mater; which is highly injected in its small vessels only; on the left side the pia mater presents a number of yellowish spots in the line of the vessels, not broader than from one to one and a half lines, and detached with the membrane; cortical substance gray-rosy; medullary moderately injected; consistence perfect; ventricles distended with about ℥iv. of troubled serosity; central parts diffluent; walls of ventricles softened to creamy consistence in a depth of from one and a half to three lines, white, surrounding injection; choroid plexus pale.—*Base.* A layer of greenish-yellow lymph, from three-fourths to one and a half lines in thickness, covered the pons, the optic and olfactory nerves, the medulla oblongata, the fissures of Sylvius, and the lateral fissures, extending through the fissures of Sylvius to the upper part of the hemisphere, and existing in a slight degree on the inferior surface of the cerebellum. This substance was beneath the arachnoid, which has a glutinous feel, and contains hardly ℥ss. of serosity. The substance had the following characters: inodorous, greenish-yellow, rather more consistence than pus, but easily broken by slight pressure without trace of granulations or other hard bodies. The cerebral substance of the base was of the natural consistence except just below the substance described, where it was whitish and pulpy. Pons varolii and cerebellum firm."

Cases judiciously treated are useful, because they teach us the use, the effects, and the value of remedies. Cases injudiciously managed are not useless, because they tell us, in intelligible accents, what we should avoid. The first are models—the second may be beacons.

Case.—*Extravasation of Blood on the Spinal Dura Mater—Death.*

The injuries of the spine have not been hitherto sufficiently investigated. Sir B. C. Brodie is engaged in their elucidation, and his judgment and experience will throw much light upon the subject. We notice the succeeding case, as an instance of serious injury of the spine, independent of fracture of the vertebræ.

A seaman, æt. 24, admitted April 26th, 1834. A short time previously he had fallen from a height, and produced a lacerated wound of the elbow-joint. He had also a scalp-wound, exposing a small portion of the cranium, and a severe contusion over the lumbar vertebræ. He was insensible for a few minutes after the fall but he then recovered; he could move his extremities without difficulty.

On the next day he had some fever.

On the next, he could not move the inferior extremities with facility.

On the third day, he complained that he could not "feel his legs," and was totally unable to move them.

On the fourth day, the paralysis of the lower extremities was complete. He complained of numbness and cramp in the uninjured arm. We should state that erysipelatous inflammation had seized upon the injured one.

On the fifth day, early in the morning, the patient was suddenly attacked with dyspnœa, anxious countenance, incapability of articulation, frequent and feeble pulse, skin cool and bathed in perspiration. The respiration became more and more laborious, and, at 9, a.m. he died.

*Dissection.* In the head, much blood is said to have existed on the exterior of the dura mater. Yet it is not stated that the blood was extravasated, and the symptoms were not indicative of such being the case.

"Muscles opposite lower dorsal and lumbar vertebræ softened; fibres scarcely perceptible, infiltrated with blood and purulent liquid; no fracture of processes. On exterior of dura mater is a layer of half-coagulated dark blood, extending throughout all the dorsal and lumbar vertebræ. Arachnoid containing a moderate quantity of serum; pia mater slightly injected. Consistence of medulla good, except about lower part of dorsal vertebræ, in extent of one and and a half inches, and in the last inch where the medullary portion is a little yellowish, and rather less consistent than elsewhere."

The lungs were gorged with blood.

No other lesions of any consequence existed.

As the injury, in this instance, was not simple, the symptoms were not distinct, nor the produce of a single lesion. It is probable that the condition of the injured arm contributed greatly to the patient's death. Yet the principal interest, at all events in our eyes, is connected with the spine.

The absence of paralysis at first, and its subsequent gradual establishment, would apparently depend on one of two causes—sanguineous extravasation slowly taking place, or inflammatory action. Effusion of blood would take place immediately on the patient's recovery from the primary insensibility—or it might be the result of secondary hæmorrhage. If the former, the paralysis would be early developed—if the latter, it would occur, like the secondary hæmorrhage, suddenly. Such are the circumstances which have been observed in the analogous case of injury of the brain, and such would probably take place in spinal injury. We say probably, for our knowledge is limited and indeterminate.

But, in the present patient, the paralysis of the extremities was not developed early, was not established suddenly, but gradually occurred after an interval of three days, and was accompanied with fever. It was apparently the paralysis dependent on inflammatory action. Now inflammation of the medulla, after injuries, usually occasions softening of its substance. The investigations of Sir B. Brodie are conclusive with regard to that. In this case, we discover diminution of consistence, and yellowness of colour in the lower inch of the dorsal medulla.

The case, we repeat, is possessed of interest.

We pass over an example of purulent deposite in the lung after amputation. We have, at various times, devoted some space to the affection, and probably we shall present a series of cases in an early Number.

The only fact remaining for notice, is an instance of phlebitis, succeeding the operation for varicose veins. We had thought that the operation was abandoned. It is difficult to determine whether it should be deemed more useless or more dangerous.

*Case.* John Farrell, æt. 30, workman in a chemical laboratory, was the third in a series of operations for varicose veins, performed by Dr. Harris in the Spring of 1834, and the only one attended by any unpleasant symptoms. The operation consisted in the removal of about three-fourths of an inch of the diseased vein, from the part that passes along the inner side of the knee.

He was admitted into hospital, on the 19th of April, for an ulcer on the right leg, accompanied with varicose veins. The operation was performed on the 27th.

"Patient did well till 30th, when he complained of pain about the knee, around which is some erysipelatous inflammation. A red line is also observed to extend upwards in the course of vein to within an inch or two of the groin, with evident thickening and tenderness on making pressure upon the part. Pulse 100, intermits every fifth beat; countenance anxious; tongue whitish; bowels not open. Ordered fifty leeches along the vein, purge with magnes. sulph.; cold mucilage to knee.

May 1st. Inflammation of vein has increased but little; patient has some fever: pulse 100, with fewer intermissions; bowels freely purged by medicine yesterday; erysipelas extending; patient much depressed; fifty leeches along vein;. followed by emplast. vesicat.; mist. neutral, ℥ss. q. 2. h."

The symptoms after this subsided gradually. It was the 1st of June before he was sufficiently recovered to be discharged.

We have nothing to say in favour of the operation. We close the present report. The facts it contains are valuable, and no doubt authentic. Yet we would venture to hint to the reporter, that a little more attention to elegance of language and a little less to trivial particulars, would materially enhance the value of his work. He seems to have imitated the tiresome verbosity of the French, who frequently write an anatomical catalogue when they ought to present a pathological digest.

---

St. Thomas's Hospital.—(bis.)

### On the Treatment of Syphilis without Mercury.

That men should disagree in matters of opinion, in inferences drawn from facts or premises, is natural and consistent with what might be expected. But that they should differ on the facts themselves, that one man should term an object white, and another affirm it to be obviously black, is a monstrous and seemingly incredible opposition.—Impossible as it might appear, the contradiction is too common to be doubted. Where interest sways, or passion blinds, the philosopher may condescend to explain and to excuse. But where these human frailties do not conspire to distort the observation and to warp the judgment, Charity herself finds it difficult to offer a favorable solution of the glaring inconsistency.

This general remark may fairly spring from a survey of the statements which amaze and confound the critic and the profession, with respect to the treatment of syphilitic symptoms. One set of observers assert that syphilis requires mercury for its removal;—the other deny that mercury is necessary, nay, they even affirm that the disease is better cured without it. Both parties found their opinions upon observation—upon *facts*. Yet some of those facts must surely lie, for Nature is true to herself. Who shall determine on which side truth is to be found?

We have had much experience in venereal maladies—have made some experiments and witnessed more. Without attempting to arrogate to ourselves the office of the umpire, we may candidly state in general terms the results of the observations we have made. Our readers will attach what importance they think fit to our conclusions, for in litigated questions of this description something must depend upon the weight of character.

We should premise that this article is based upon two papers; one by Dr. Williams, of St. Thomas's Hospital—the other by Dr. Green of Bristol. The first is contained in the Medical Gazette;* the second will be found in the second volume of the Provincial Medical Transactions.

The great point d'appui of the anti-mercurialists is the army medical reports. Those reports display a formidable battery, from which the mercurial practice is assailed. Perhaps the most forcible statements in those documents, are contained in the following extract from the paper of Dr. Green.

"The reports I have next to call attention to, were circulated from the Army Medical Department, in April, 1819, and will be found in Dr. Hennen's work; they state as follows:—'That since Dec. 1816, to Dec. 1818, there appear to have been treated, for primary venereal ulcerations on the penis, (including not only the more simple sores, but also a regular proportion of those with the most marked characters of syphilitic chancre, as described by Hunter and other writers) 1940 cases.' 'That of these 1940 cases so treated, 96 have had secondary symptoms of different sorts;' of these 96 cases of secondary affections, mercury was had recourse to in 12, for various reasons stated in the report. In the 1940 cases of primary symptoms, mercury was used in 65, for reasons also assigned; if we deduct the 65 and 12 cases in which mercury was used, from 1940 reported, 1863 *cases remain completely cured without mercury.* The report goes on to state, 'that the average period required for the cure of primary symptoms without mercury, when bubo did not exist, has been 21 days; with bubo, 45 days.' That the average period for the cure of secondary symptoms, without mercury, has been from 28 to 45 days. '*That every man treated without mercury, has been fit for immediate military duty on dismissal from the hospital.*' In the same period, 2827 cases of primary symptoms were treated with mercury; secondary symptoms occurred in 51 of them. The report states that, in the majority of these instances, there were good grounds for believing that *the constitutional symptoms 'were more severe and more intractable than when mercury had not been used for the primary sores.'* Two more thus treated

* Med. Gaz. April 12th, 1834.

*with mercury*, were discharged the service, *on account of the injury their constitutions sustained from the treatment.* The average period required for the cure of primary symptoms, without bubo, was 33 days; with bubo, 50 days; and for the cure of secondary symptoms, 45 days. *Here, then*, we have *a record of* 1833 *cases, of every form and stage of syphilis, cured without mercury;* and in not one instance did any unfavourable results follow the treatment; foul ulcerations, caries of the bone, broken down constitutions, and a loathsome death, were not here to be seen. The evidence thus presented is too strong to be disputed, and, perhaps, could have been collected from no other source than military hospitals, and in no other practice could the consequences of the treatment be better observed, as, of course, the men remain under the personal observation of their surgeons after they are cured. I need hardly say, that the enquiries were conducted with that fair, candid, and impartial spirit, which should ever mark the investigations of men searching for truth; if any triumph did follow their exertions, it was that of truth over error."

When we add to this the result of the experiments of Mr. Rose, we have laid before our readers the leading statements contained in the medical reports of the army—statements to which the anti-mercurialists constantly refer with confidence and triumph—the triumph, as they tell us, of truth over error.

" The late Mr. Rose, surgeon to the Coldstream Guards, first stated to the profession in this country,* the important fact, that all cases of the primary and secondary symptoms of syphilis could be cured without mercury. For a year and three quarters he treated, at the regimental hospital, in London, all cases of venereal diseases occurring in the soldiers of the Coldstream Regiment of Guards, without mercury; he observes,† 'I have certainly succeeded in curing all the ulcers of the parts of generation, which I have met with in that period, with the constitutional symptoms to which they give rise, without the exhibition of mercury.'—Mr. Rose treated more than 120 cases in this way, and no unfavourable results whatever followed the practice; he adds,‡ 'caries of the bone, and some of the least equivocal symptoms did not occur. Constitutional symptoms were, in most instances, mild, and, in some, so slight, that they would have escaped notice unless carefully sought for.' In not one instance was there that uniform progress, from bad to worse, which was considered an essential characteristic of true syphilis. Mr. Rose's paper is well worthy of attentive perusal, as well on account of the facts it contains as of the spirit of cautious enquiry evinced in it. Mr. Guthrie next published a paper on the same subject; he had treated nearly a hundred cases of primary sores without mercury, and considers it an established fact, that§ 'every kind of ulcer of the genitals, of whatever form or appearance, is curable without mercury.' He considers that, in some cases, a gentle course of mercury will expedite the cure, but does not consider it a specific for the venereal disease."

Dr. Green, of Bristol, who quotes and who admires the foregoing reports—who endeavours

To swell the triumph and partake the gale, has his own experience to confirm their accuracy. His modesty induces him to suppose that his testimony is feeble, yet his candour compels him to present it, in the hope and with the object of inducing surgeons to regard the anti-mercurial treatment without distrust.

For the last seven years, he has

* " Mr. Furguson had previously stated, in the 4th vol. of the Med. Chir. Trans. that the venereal disease was successfully treated in Portugal, without mercury."

† Medico-Chirurgical Transactions, vol. viii. p. 355.

‡ Med. Chir. Trans. vol. viii. p. 420.

§ Ibid. p. 571.

treated nearly all the cases of venereal that came before him, without mercury. In a few instances, indeed, he has employed this remedy, where the symptoms got into an indolent or chronic state. He "tried the stimulus of mercury" in such cases, precisely as he would have done whether the symptoms were of venereal origin or not, with the view of inducing healthy action in the local disease; or he has given it to alter the secretions from the stomach and bowels, or to improve the general health where it seemed required.

"I have kept an account of 100 cases of the venereal disease, treated by myself, without a particle of mercury used internally or externally. In the selection of these cases, two objects have been kept in view, namely, that the primary sore should, in some degree, possess the characters of the Hunterian chancre, 'a circular, depressed, and sloughy sore, with an indurated base;' and I have preferred taking notes of those cases where patients resided in Bristol, that I might have an opportunity of watching the consequences of the treatment, to ascertain if any symptoms of a contaminated constitution should result from syphilis, cured without its supposed specific."

Our crippled space will permit us to offer no more than a summary of our author's facts—a still more brief account of his opinions.

And first of his facts.

1. All the primary sores exhibited more or less resemblance to the Hunterian chancre. They were treated with sedative and astringent lotions, or with simple ointment as one or the other best agreed. The average period which they took to heal was a fortnight to a month. In some of the cases, the local inflammation accompanying the sore required local or general blood-letting; in others, thickening and induration remained for some time after the sores were cicatrized, but spontaneously subsided.

2. Of 100 cases of primary venereal sores thus treated, buboes supervened in sixteen. Of these buboes, six suppurated. The buboes which did not suppurate, were removed, on an average, in about six weeks; in two cases, however, a chronic enlargement of the inguinal glands remained for a longer period, one four, and the other seven months; but both of these patients were of decidedly scrofulous habits. The buboes which did suppurate, were healed within two months from their appearance, except one, which remained open four or five months; this patient was also of a scrofulous constitution; ultimately, the whole sixteen cases of bubo were cured.

3. Constitutional affections, of one kind or another, followed in nine cases; these secondary symptoms were, cutaneous eruptions, sore throat, pains in the limbs and periostitis. The affection of the skin assumed varied forms; it was papular in three cases, pustular in two, vesicular in one, vesicular and scaly in two, and in the last it was a mixed form of eruption, papular in the beginning, followed by a general redness, and ending in a scaly condition of the surface. Two of the papular eruptions were so slight, as scarcely to deserve attention. One was a well-marked specimen of "lichen simplex;" it lasted about three weeks. One case of pustular eruption displayed a striking resemblance to small-pox;—in four months it was removed. The two cases of vesicular and scaly eruptions occurred in delicate and strumous persons; one lasted between three and four months—the other nearly seven. The mixed form of eruption was gone in five weeks.

4. Sore throat occurred in four cases; in three it was conjoined with cutaneous eruptions. We will only observe of the ulceration of the throat that it was extensive, superficial, and covered on its surface with a thin layer of adherent lymph.

5. Periostitis occurred in two cases—broadly marked in one, and faintly in the other. In one, the bones of the head were affected; the complaint was obstinate, but yielded at length to counter-irritation. In the other the tibia was the bone attacked; no treatment was required in this case.

6. There was not one instance of iritis.

Dr. Green deems it necessary to mention the unfavourable symptoms that were noticed. They are neither numerous nor grave.

"In one case of tedious cutaneous eruption, the chain of lymphatic glands of the neck became swollen, and they have remained enlarged since. In another case of obstinate scaly and vesicular eruption, the skin remained discoloured, in patches, for nearly twelve months."

Such are the facts of Dr. Green. His opinions may be analytically reduced to this:—that it is absurd and false to consider mercury a specific for syphilis. The belief in its specific agency is, he thinks, only worthy of a place in the manual of some Turkish Doctor, or the hoarded secrets of some copper-coloured Indian. He denies that mercury has any power whatever over the syphilitic poison itself—a theory, he observes, unsupported by facts, which has never yet been proved, and never will be. Warming as he proceeds, he throws down the gauntlet against *all* specifics; "a belief in which is to say the least of it, a very unscientific faith."* To sum up—he conceives that, cæteris paribus, the venereal disease can be better cured without mercury than with it—that worse consequences follow when the latter is employed than when rejected—and, therefore, that its use should in general be abandoned. Yet he owns that it is sometimes beneficial—but *not* as a specific; his hypothesis and facts continue safe, his creed is not alarmed, at exhibiting the mineral, *not* as a specific.

"The cases in which mercury may be employed with advantage, appear to me to be those in which the symptoms get into an indolent state, and become a chronic disease. Here mercury may be of service, if there be nothing in the constitution of the patient to forbid its use. Its chance of doing good in these cases, results not from any specific or antisyphilitic power belonging to the remedy, but from the well-known influence mercury possesses of controlling and altering morbid actions, altogether devoid of any specific character; but, if there be any difference between its influence over specific diseases, and those of a different nature, experience tells us it is this, that while mercury alters and subdues simple morbid actions, it alters and sometimes aggravates those of a specific nature, such as the phenomena resulting from the venereal infection."

Dr. Williams comes next upon the stage. If Dr. Green has prepared the bane, Dr. Williams presents the antidote—if the former denounces mercury as a specific—the latter considers its powers undeniable.

His arguments in favour of those powers are brief—they are general deductions, rather than circumstantial statements.

1. Dr. Williams admits, as it is impossible to deny, that syphilitic, that is venereal, sores, and, indeed, venereal secondary symptoms, will in certain instances terminate spontaneously. But he contends that, where mercury is not given, the disease is much prolonged, and secondary symptoms are more frequent.

"It is necessary, also, to ensure a favourable result, that the patient should submit to a painfully long confinement to his bed, and not only that he should abstain from every indulgence, but must submit to an exceedingly low or spoon diet. A spontaneous cure, therefore, is a termination little in unison with our habits of active life, and some remedial mode of treatment is not merely desirable but necessary. In the cure,

* The Doctor's notions and our's on science may, probably, be widely different. His science may consist in something intellectual, metaphysical, and abstract—our's is the homely induction from fact; his goddess may be throned in the æther or the clouds—our's walks amidst the haunts of men; he may deem it unscientific to believe that sulphur is a specific for the itch—our science is unable to dispute or to contemn the humble truth.

then, of syphilis, mercury in greater or less doses, and introduced into the system either locally or by the mouth, or by inunction, is unquestionably, according to our present experience, the great, and indeed only specific remedy by which we are enabled to control and subdue the primary action of this baneful poison."

2. The cure of the secondary symptoms Dr. Williams thinks to be a problem. Yet he makes a faint attempt at its solution.

"The problem of the cure of the secondary symptoms is not of such easy solution. It has been affirmed that these symptoms are aggravated wherever mercury has been used for the cure of the primary sores: of this, however, I entertain the strongest doubts; for it is agreed that secondary symptoms much more frequently occur when the primary symptoms have been allowed to terminate spontaneously than when mercury has been used, and yet it seldom happens, when there is so strong a tendency to disease, that such disease, when excited, is mild. It is a fact also, that before mercury was used for the cure of syphilis, it took its name from the more hideous of the forms of its secondary symptoms. In Lisbon also, where mercury is little used for the primary sores, every military surgeon admits that more mutilated faces are to be seen than in any other town of the same size in Europe. But whatever may be the fact, it is certain that the cases of secondary symptoms which present themselves at the London hospitals are of great severity, and shew little tendency to a spontaneous cure. On the contrary, the long and extreme sufferings of the patients, and their worn and emaciated frames, demand prompt and immediate relief. In the cure then, of the secondary symptoms, mercury and sarsaparilla are the only remedies that at present maintain any character.

The medicinal properties of the latter substance, however, are so little determined—the cases in which it is useful so little agreed on—that Mr. Hunter, and also many most eminent physicians and surgeons, have abandoned it as altogether inefficient; and it is hardly an exaggeration to state that the majority of the profession, in the present day, rely on mercury as the only remedial agent in all forms of the disease.

If we look, however, to the laws which govern other poisons, we find that their different specific actions are only subdued by many and even opposite remedies; and consequently we might expect that although mercury might be eminently useful in some stages of syphilis, still, that in combating so multiform a disease, many different modes of treatment would be necessary. In paludal disease, for example, as long as the action of the poison is limited to inducing intermittent fever, unaccompanied by any local disease, quinine is a certain and specific remedy. But no sooner are the secondary affections of that poison established, as inflammation of the liver or spleen, than calomel is the specific medicine; quinine being either unavailing or injurious. An equally remarkable change of treatment is required should the poison of scarlet fever fall upon the larynx or peritoneum, or on the synovial membrane of the joints. It will be plain, therefore, that mercury, the great specific in the primary affections of syphilis, is of doubtful efficacy in its secondary affections; and every practical physician knows how often this powerful remedy totally fails in relieving many of the most distressing forms of the disease."

Such are the arguments adduced by Dr. Williams, in his general support of a mild mercurial practice. The remainder of his paper is occupied with some reflections upon nodes and venereal ulceration of the throat. Our business is at present with the question of exhibiting or withholding mercury for the cure of syphilis. We must, therefore, disregard the observations of the Doctor, which have not particular reference to this subject. Yet we cannot agree with some of his opinions, and his paper may furnish, at a future opportunity, the peg on which to hang some critical remarks.

When we look at the present condition of the argument, we find that it assumes the following aspect. On one side is displayed an alluring and imposing array of facts—cases in the mass of a specious character, which subdue the reàsoner by their demonstrative appearance. On the other hand, we discover the majority of the profession, and its experienced portion in particular, clinging, perhaps with prejudice, but certainly with pertinacity, to the doctrine which those facts are calculated to subvert. Cherished errors and prejudices are strong, but truth, after a time, is stronger; and it does appear to offer primâ facie evidence in favor of mercury, that the anti-mercurialists so far from gaining ground are daily losing it.

There are reasons for believing *that* a keen examination of the Army Medical Reports would not lead the impartial reasoner to conclusions so unfavourable to the value of mercury, as Dr. Green and others believe; *that* mercury was resorted to in cases which had resisted the non-mercurial treatment; *that* out of a certain number of sores promiscuously taken many will not be syphilitic at all; *that* if sores be not syphilis, mercury given as a specific would be mischievous, and, consequently the comparison of numbers with numbers treated each way is not a fair one; the point at issue being mercury or no mercury for syphilis only; *that* the experiments were made by men, some, at least, of whom were ill-informed, both of the forms and nature of syphilis, and the proper mode of exhibiting mercury; men with no hospital experience, an imperfect education, and the liabilities to error arising from the enthusiasm excited by the support of a novel doctrine; *that* with all these disadvantages—the application of mercury in cases in which it should not be applied, its exhibition in proper cases in an improper manner, the necessary incompetence of many of the experimenters to conduct such vast and such nice experiments, and all the fallacies which we will not name—with all these disadvantages, the careful consideration of the whole of the reports will still leave the balance in favor of mercury.

Perhaps the investigations of the late Mr. Rose were less likely to terminate in error than those of many of his associates. Those investigations are constantly appealed to as decisive evidence against mercury. Mr. Rose became surgeon to St. George's Hospital, and we can state with confidence, for many gentlemen know the fact, that he *abandoned* in later life the non-mercurial treatment—that he confessed its inefficiency—and employed the remedy which his statements are frequently brought forward to condemn. We may mention an instance, which came, among others, to our knowledge. A young physician contracted a sore upon the prepuce. He went to Mr. Rose. The latter gentleman merely observed, "I would advise you to take a course of mercury." "Oh! but," said the Doctor, "it can be cured without it." "Never mind that," replied the anti-mercurialist, "if you take my advice you'll take mercury." Mr. Rose was an honourable man, and would never have published what he did not believe to be true. He reluctantly changed his opinions, but he did change them.

Mr. Guthrie is adduced as another authority against the use of mercury. We would ask this question:—*does* Mr. Guthrie now pursue the non-mercurial practice? We should fancy that he does not.

The length to which this article has stretched prevents us from offering many observations arising from our own experience. Yet that experience has not been narrow in the venereal disease. In our next number we shall make some more extended remarks upon the subject.

The facts which we have witnessed, and the experiments which we have made have been sufficient to convince us of the utility, nay necessity, of mercury. Like many others, we were charmed with the plausibility of the Army Medical Reports—we imagined that the time was come when syphilis could be treated safely and judiciously without a course of mercury. We were soon undeceived. The great opportunities afforded for observation and for practice in the wards of the Lock Hospital speedily disclosed to us the important truth, that whatever might be the case with respect to the venereal disease elsewhere, no doubt could be entertained of its being best cured by mercury *there*. We do not disbelieve the army surgeons, we do not dispute the authority of Dr. Green, but we believe the evidence of our own senses, and that evidence is in favour of mercury.

Our observations may be reduced to the following general statements:—*that* sores possessing the characters of syphilis, when treated without mercury, are remarkably prone to occasion secondary symptoms — *that* secondary symptoms, treated without mercury, are in general extremely obstinate, and in many cases appear to promise no natural termination—*that*, upon the other hand, the same kind of sores and the same description of secondary symptoms, yield with comparative facility to mercury, judiciously exhibited—*that* some sores require but little mercury, and some probably require none—*that* some symptoms usually reputed secondary, such as rupia, sloughy ulceration of the throat, and nodes, are occasionally benefited, and as often aggravated by the employment of mercurial medicines—and, finally, *that* mercury, properly administered, is not only valuable, but, practically speaking, indispensable for lues. Such are the conclusions we have drawn from observation and experience, in the Lock Hospital and out of it. That observation has been minute and diligent, if it has not been successful; and, without attempting to play the braggart, we will say that our experience has not been inconsiderable. We went to the investigation with a bias against mercury; the inquiry has given birth to a conviction for it.

The fact is, that there is much, very much, to be done in the examination of the venereal disease. We possess no really good work upon the subject. Hunter's is mischievously wrong—the more modern productions are less boldly erroneous. Many syphilitic symptoms are not at all, or imperfectly delineated—other symptoms are described as syphilitic, which are dubious in their origin or character. Mercury is either not used, or it is abused—it is withheld when it would be safety, given when it is destruction. Scylla and Charybdis are on either side, and to avoid the one, the profession too commonly rush upon the other. In short, venereal symptoms are not sufficiently discriminated, mercury is not appropriately exhibited, and the result, as might reasonably be expected, is confusion.*

---

* In the foregoing article, want of space has compelled us to omit our facts, and to mutilate our opinions. In a succeeding Number, we shall endeavour to point out the symptoms that require, and the best mode of giving mercury.

